# RIN4 Functions with Plasma Membrane H^+^-ATPases to Regulate Stomatal Apertures during Pathogen Attack

**DOI:** 10.1371/journal.pbio.1000139

**Published:** 2009-06-30

**Authors:** Jun Liu, James M. Elmore, Anja T. Fuglsang, Michael G. Palmgren, Brian J. Staskawicz, Gitta Coaker

**Affiliations:** 1 Department of Plant Pathology, University of California Davis, Davis, California, United States of America; 2 Centre for Membrane Pumps in Cells and Disease—PUMPKIN, Danish National Research Foundation, Århus and Copenhagen, Denmark; 3 Plant Physiology and Anatomy Laboratory, Department of Plant Biology, University of Copenhagen, Frederiksberg, Denmark; 4 Department of Plant and Microbial Biology, University of California Berkeley, Berkeley, California, United States of America; Max Planck Institute for Developmental Biology, Germany

## Abstract

Pathogen perception by the plant innate immune system is of central importance to plant survival and productivity. The *Arabidopsis* protein RIN4 is a negative regulator of plant immunity. In order to identify additional proteins involved in RIN4-mediated immune signal transduction, we purified components of the RIN4 protein complex. We identified six novel proteins that had not previously been implicated in RIN4 signaling, including the plasma membrane (PM) H^+^-ATPases AHA1 and/or AHA2. RIN4 interacts with AHA1 and AHA2 both in vitro and in vivo. *RIN4* overexpression and knockout lines exhibit differential PM H^+^-ATPase activity. PM H^+^-ATPase activation induces stomatal opening, enabling bacteria to gain entry into the plant leaf; inactivation induces stomatal closure thus restricting bacterial invasion. The *rin4* knockout line exhibited reduced PM H^+^-ATPase activity and, importantly, its stomata could not be re-opened by virulent *Pseudomonas syringae*. We also demonstrate that RIN4 is expressed in guard cells, highlighting the importance of this cell type in innate immunity. These results indicate that the *Arabidopsis* protein RIN4 functions with the PM H^+^-ATPase to regulate stomatal apertures, inhibiting the entry of bacterial pathogens into the plant leaf during infection.

## Abstract 2

In plants, the protein Rin4 acts with the plasma membrane H^+^-ATPase to regulate pathogen entry and the innate immune response, in part, through the regulation of stomatal closure.

## Author Summary

Plants are continuously exposed to microorganisms. In order to resist infection, plants rely on their innate immune system to inhibit both pathogen entry and multiplication. We investigated the function of the *Arabidopsis* protein RIN4, which acts as a negative regulator of plant innate immunity. We biochemically identified six novel RIN4-associated proteins and characterized the association between RIN4 and the plasma membrane H^+^-ATPase pump. Our results indicate that RIN4 functions in concert with this pump to regulate leaf stomata during the innate immune response, when stomata close to block the entry of bacterial pathogens into the leaf interior.

## Introduction

Plants are continuously exposed to a variety of microorganisms. In order to successfully avoid infection, they have evolved a series of defense mechanisms that work in concert to limit pathogen invasion and multiplication [1]. Unlike vertebrates, plants lack an adaptive immune system and rely on their innate immune system to recognize and restrict pathogenic microbes. Conceptually, there are two primary branches of plant innate immunity. One branch employs extracellular receptors to recognize conserved microbial features termed pathogen-associated molecular patterns (PAMPs), resulting in PAMP-triggered immunity (PTI). The second branch uses intracellular plant resistance (R) proteins to recognize pathogen effectors delivered inside host cells during infection, resulting in effector-triggered immunity (ETI). Despite the importance of plant innate immunity, how pathogen perception activates immune responses and signaling overlap between PTI and ETI remain elusive.

PAMPs are conserved microbial features, such as bacterial flagellin or fungal chitin, which fulfill a function crucial to the lifestyle of the organism. PAMPs are perceived by pattern-recognition receptors resulting in PTI. The activation of PTI leads to the induction of mitogen-activated protein kinase (MAPK) signaling, transcriptional reprogramming, production of reactive oxygen species, and callose deposition, which serves as a physical barrier at infection sites (reviewed in [2]).

In order to colonize plants, virulent microorganisms need to overcome PTI. Plant pathogenic bacteria use the type III secretion system to deliver 20–30 effector proteins into the plant cell during pathogenesis. Collectively, these effectors are required for virulence and individual effectors have been shown to inhibit PTI through a variety of mechanisms [3]. The most well-studied bacterial effectors come from *P. syringae* pv. *tomato* (*Pst*), the causal agent of bacterial speck on *Arabidopsis* and tomato. In susceptible plant genotypes effectors enhance pathogen virulence and can inhibit PTI and ETI; in resistant plant genotypes effectors are recognized, culminating in an inhibition of pathogen growth [4],[5]. Despite the wide range of pathogens recognized, the majority of R genes can be grouped into one large family encoding proteins with a nucleotide-binding site (NB) and C-terminal leucine rich repeat (LRR) domains [6]. Several plant R proteins can detect effectors indirectly by monitoring for effector-induced perturbations of key host proteins.

To date, RIN4 (At3g25070) is the only known protein that can regulate both branches of the plant immune system. *RIN4* overexpression lines exhibit decreased callose deposition after PAMP treatment as well as enhanced growth of virulent and type III secretion-deficient *Pst*, indicating a reduction in PTI [7]. *rin4* knockout lines exhibit increased callose deposition after PAMP treatment and decreased *Pst* growth, consistent with enhanced PTI signaling [7]. These data indicate that RIN4 is a negative regulator of PTI. In addition, two R proteins, RPM1 (At3g07040) and RPS2 (At4g26090), monitor RIN4. RPM1, RPS2, and RIN4 are all localized to the plasma membrane [8]–[10]. In the absence of pathogen perception, RIN4 acts as a negative regulator of RPM1 and RPS2. When the *P. syringae* effectors AvrRpm1 or AvrB are delivered to the plant cell, RIN4 is hyper-phosphorylated, which in turn leads to the activation of RPM1-mediated resistance [8]. Another *P. syringae* effector, AvrRpt2, is a protease that directly targets RIN4, leading to the activation of RPS2-mediated resistance [11]–[14]. Investigation of the *Arabidopsis–P. syringae* interaction has identified RIN4 is a point of convergence for the regulation of both PTI and ETI. However, a mechanistic understanding of how RIN4 negatively regulates PTI remains elusive.

Many pathogenic bacteria can proliferate as epiphytes on the plant leaf surface, but in order to infect a plant they must colonize host tissues. Bacterial pathogens gain entry inside plant leaves through wounds or natural openings like stomata. Stomatal pores, located on the aerial epidermis, permit gas exchange between plants and the atmosphere. A pair of guard cells surrounds stomatal pores. Guard cells respond to diverse stimuli in order to regulate stomatal apertures including: blue light, temperature, humidity, CO_2_, plant hormones, and pathogen inoculation [15]–[17]. Stomatal pores operate as osmotic machines that open when the PM H^+^-ATPase of guard cells is allowed to be active. The activity of this proton pump generates a large transmembrane electrochemical gradient that drives the uptake of charged solutes and, as a consequence, water, which in turn causes the cells to swell and the pore between them to open. Stomatal closure is initiated upon depolarization of the guard cell plasma membrane by inhibiting the PM H^+^-ATPase.

Historically, stomata were thought to be passive ports of entry, but recent evidence reveals that stomatal closure is induced by both PTI and ETI in an attempt to restrict bacterial invasion [15],[18],[19]. Upon perception of PAMPs, stomata will close within 1 h. However, virulent bacteria are able to re-open stomata after 3 h, facilitating their entry into the plant leaf. For example, virulent *Pst* secretes the polyketide toxin coronatine, which stimulates the plant to re-open their stomata [15],[20]. Several other pathogenic microorganisms also act to regulate stomatal apertures during infection [19],[21]–[23]. One particularly well-characterized example is the toxin fusicoccin, produced by the fungal pathogen *Fusicoccum amygdali* [24]. Fusicoccin is a strong activator of the plasma membrane H^+^-ATPase and rapidly induces stomatal opening, presumably in order to facilitate fungal penetration [25]–[27]. Taken together, these data highlight the importance of stomatal pores and guard cell signaling during pathogen infection.

In this study, we report the identification and characterization of the *Arabidopsis* RIN4 protein complex. We were able to purify several associated proteins by immunoaffinity chromatography and identify them by mass spectrometry. We identified the PM H^+^-ATPases AHA1 (At2g18960) or AHA2 (At4g30190), whose interaction we characterized in greater detail. The C-terminal regulatory domain of AHA1 and AHA2 interact with RIN4 by yeast two-hybrid and we can detect a specific interaction between AHA1/AHA2 and RIN4 in planta using bimolecular fluorescence complementation (BiFC). *RIN4* overexpression enhanced PM H^+^-ATPase activity, while the *rin4* knockout line exhibited decreased PM H^+^-ATPase activity. Importantly, we demonstrate that the *rin4* knockout cannot re-open its stomata in response to virulent *Pst*. We also show that *RIN4* is expressed in guard cells along with other PTI and ETI signaling components. Our findings are consistent with a model in which RIN4 associates with the C-terminal autoinhibitory domain of the PM H^+^-ATPase to regulate leaf stomata in response to PAMPs.

## Results

### Purification and Identification of the RIN4 Protein Complex

In order to gain a more comprehensive understanding of the proteins involved in plant immune signaling, we investigated the components of the RIN4 protein complex in *Arabidopsis thaliana*. We used affinity-purified antibody recognizing RIN4 to purify associated proteins by immunoaffinity chromatography (Figure S1). The *rps2-101c* mutant complemented with the RPS2 transgene containing a C-terminal fusion to the hemagglutinin (HA) epitope was used for RIN4 purifications. This line is biologically relevant because RPS2:HA is expressed from its native promoter, can complement the *rps2-101c* mutation, and confers resistance to *Pst* expressing AvrRpt2 [11]. RPS2 associates with RIN4 in planta, and we used this association to troubleshoot purification conditions. Because the *rin4* knockout is lethal in the presence of RPS2, we used the *rps2/rin4* double mutant line to control for nonspecific protein binding [13]. Multiple purification protocols were tested in order to identify conditions that would enable us to detect the presence of both RIN4 and RPS2 by mass spectrometry. We found that wash conditions containing more than 150 mM NaCl eliminated most nonspecific protein binding, but also eliminated our ability to copurify RPS2 in the positive controls. Protein complex purifications were also conducted after plasma membrane fractionation, but this eliminated our ability to copurify RPS2 (unpublished data). Therefore, we used whole leaf protein extracts and mild wash conditions to purify RIN4 associated proteins across three biological replicates. Proteins from each sample were analyzed directly using high performance liquid chromatography coupled to tandem mass spectrometry (MS; Figure S1). Proteins were identified using the MASCOT algorithm to search the *Arabidopsis* genome. All experiments captured native, biologically relevant levels of RIN4 and associated proteins.

We reproducibly identified RIN4 and RPS2 as well as six novel RIN4-associated proteins across three biological replications (Tables 1, S1, and S2). In order to be classified as a RIN4-associated protein, the protein had to be identified by a minimum of two unique peptides and be present in all three replications of the positive control, but never identified in the negative control *rps2/rin4*. Although we were able to identify RPS2 and RIN4 by mass spectrometry, we did not identify two additional proteins that are known to interact with RIN4: NDR1 (At3g20600) and the R protein RPM1 [8],[28]. Both proteins have been demonstrated to interact by yeast two-hybrid and co-immunoprecipitation. Our inability to detect RPM1 could be because only a small percentage of RPM1 interacts with RIN4 in the plant, indicating that these two proteins may transiently interact during ETI [8]. Alternatively, our mass spectrometry analysis may have only identified the most abundant RIN4 associated proteins. In contrast to RIN4, which is easily detected by western blot, RPM1 and NDR1 are expressed at very low levels, making them difficult to identify by mass spectrometry.

**Table 1:** Members of the RIN4 protein complex identified by mass spectrometry.

Protein	Gene Identifier	Accession Number (IPI)	*rps2 RPS2:HA* (1)	*rps2 RPS2:HA* (2)	*rps2 RPS2:HA* (3)
RIN4	At3g25070	IPI00517440	7	9	8
RPS2	At4g26090	IPI00547830	0	3	2
AHA1 or AHA2	At2g18960 At4g30190	IPI00526113	13	8	4
ERD4	At1g30360	IPI00526219	5	4	2
Remorin	At3g61260	IPI00539947	2	3	2
MATH domain	At3g28220	IPI00528031	2	6	12
Jacalin domain	At1g52000	IPI00531879	2	2	2
Jacalin domain	At3g16420	IPI00543838	2	6	6

Counts are the sum of three biological replications (except RPS2, identified in only two replications). Protein identification required *p*<0.05 (MOWSE algorithm), minimum two peptides. We were unable to differentiate between AHA1 and AHA2 by mass spectrometry during runs 2 and 3, two PM H^+^-ATPases. The number of unique peptides identified for each protein is listed. None of these proteins were identified in the negative control (*rps2-101c/rin4* knockout line).

A MATH domain protein, two Jacalin domain proteins, ERD4, a remorin, and the PM H^+^-ATPases AHA1 and/or AHA2 were identified by mass spectrometry (Tables 1 and S1). The MATH domain is broadly represented in eukaryotes [29]. Proteins containing MATH domains, primarily the well-characterized *TNF Receptor Associated Factor* family, are involved in human disease resistance signaling through their regulation of inflammation and apoptosis responses [30]. MATH domains are thought to act as protein adapters, transferring signals to intracellular signaling pathways. Proteins containing MATH domains are prevalent throughout the plant kingdom, but have not been characterized or implicated in plant disease resistance. Jacalins are lectins, which have been shown to be induced in response to the hormone methyl jasmonate [31]. *ERD4* (Early Responsive to Dehydration 4) was originally identified because it is rapidly induced during drought stress [32]. Microarray analysis has revealed that *ERD4* is also induced in response to multiple biotic and abiotic stresses, although its function remains elusive (unpublished data). Remorins are plasma membrane associated proteins of unknown function with C-terminal coiled-coiled domains. Multiple remorins possess an N-terminal domain with similarity to viral movement proteins [33]. All of these proteins are predicted to be membrane-localized, which is where RIN4 resides [8].

We also identified the PM H^+^-ATPase (AHA), the proton pump responsible for energization of the plasma membrane. We were unable to distinguish between the highly homologous AHA1 and AHA2 proteins by mass spectrometry in two out of three biological replications. We were able to identify AHA1 specific peptides in the first MS run (Table S2). There are 11 *AHA* genes in *Arabidopsis*, which pump H^+^ from the cytosol to the apoplast in an ATP-dependent manner. *AHA1*, *AHA2*, and *AHA5* are the major transcripts found in guard cells [34]. AHA1 and AHA2 are predicted to have molecular masses of 104.2 and 104.4 kDa, respectively, and share 94% amino acid identity. In light of recent data implicating *AHA1* in stomatal regulation and the role of stomatal closure in the innate immune response, we decided to analyze the association between RIN4 and AHA1/AHA2 in greater detail [15],[35].

### The PM H^+^-ATPase Interacts with RIN4 by Yeast Two-Hybrid and In Planta

**Figure 1:**
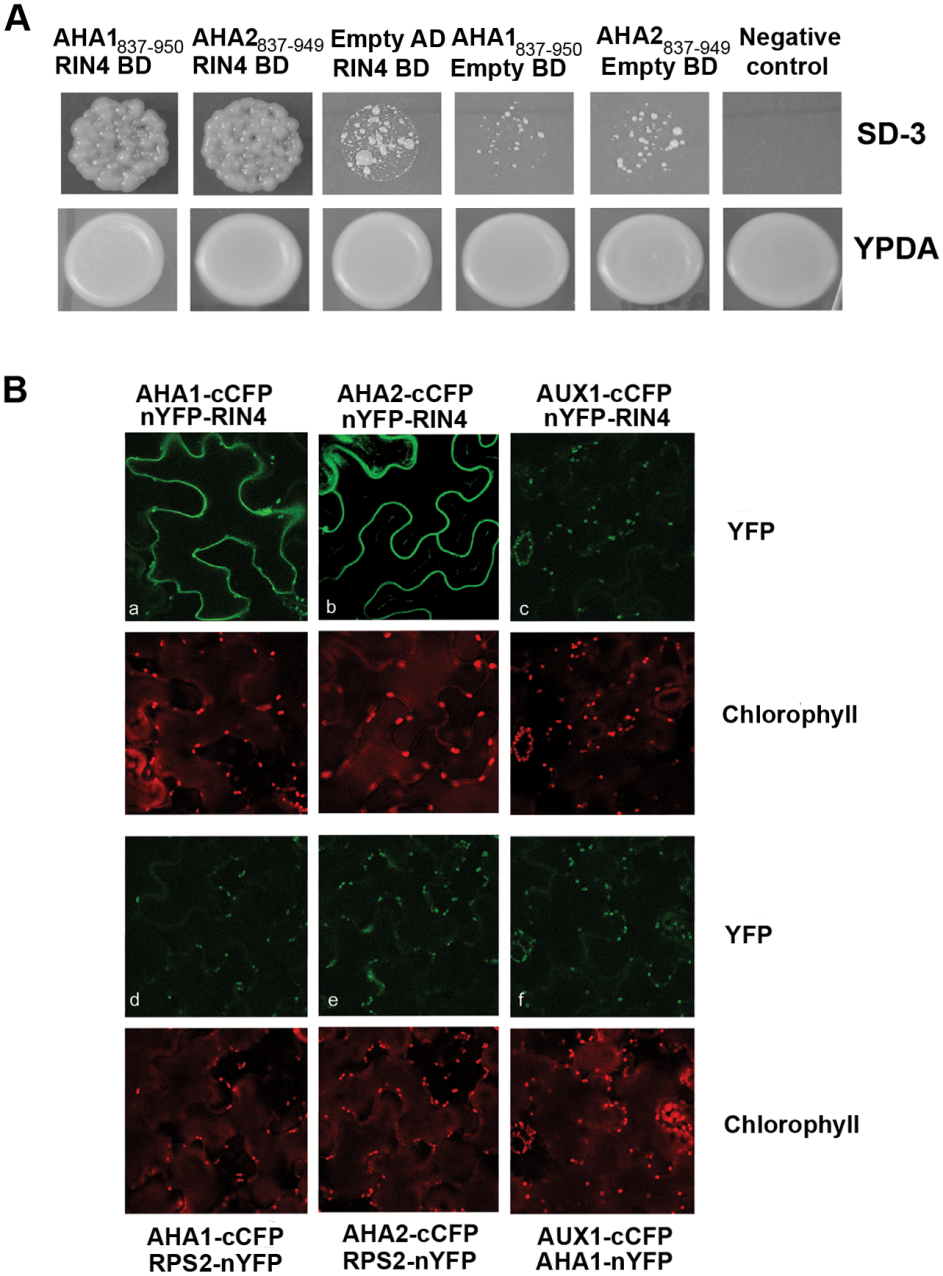
RIN4 and AHA interact in planta and in yeast. (A) RIN4 interacts with the C terminus of both AHA1 and AHA2 using the Matchmaker yeast two-hybrid system (Clontech). BD, binding domain vector (pGBKT7); AD, activation domain vector (pGADT7); SD-3, synthetic dextrose media lacking leucine, tryptophan, and histidine; YPDA, yeast potato dextrose agar. AHA1 and AHA2 were cloned into the AD vector. (B) AHA1 and AHA2 associate with RIN4 in vivo. We were able to detect a specific interaction between AHA1 and AHA2 with RIN4 by BiFC across three replications. The experiments to detect BiFC fluorescence with AHA1 and AHA2 were conducted independently. *N. benthamiana* leaves co-expressing either AHA1 or AHA2 and RIN4 results in detectable GFP fluorescence on the membrane (upper panel, a–b). No interaction with RPS2 (lower panel, d–e) or AUX1 (c, f) could be detected. YFP fluorescence was excited at 488 and imaged at 518–540 nm by confocal microscopy, except for b and e (used excitation 512 and emission 525–540 nm). Chlorophyll emission was detected at 618 nm.

In order to validate the RIN4 AHA1/AHA2 association detected by mass spectrometry, we subjected them to BiFC and yeast two-hybrid analyses. AHA1 and AHA2, which are negatively regulated by their C termini, possess multiple transmembrane domains (reviewed in [36]). Therefore, we employed the hydrophilic C-terminal regulatory domain of AHA1 and AHA2 in our yeast two-hybrid analyses. As shown in Figure 1A, we detected an interaction between RIN4 and the C termini of both AHA1_837–950_ and AHA2_837–949_, when compared with the negative control T-antigen/Lamin-C using the Matchmaker system. We were unable to detect any interaction between RPS2, AHA1_837–950_, or AHA2_837–949_ by yeast two-hybrid (unpublished data). We verified that RIN4, AHA1_837–950_, and AHA2_837–949_ are expressed in yeast and do not autonomously activate His auxotrophy (Figures 1A and S2). We also tested beta-galactosidase activity, but could only detect a faint blue color (unpublished data). These results indicate that RIN4 can weakly interact with the C terminus of AHA1 and AHA2 by yeast two-hybrid.

To provide additional evidence for the AHA and RIN4 interaction, we investigated the association between AHA1, AHA2, and RIN4 in planta using a BiFC approach to directly visualize protein interactions in living cells. A specific interaction between either AHA1 or AHA2 and RIN4 was detected in *Nicotiana benthamiana* leaves (Figure 1B, a, b). The yellow fluorescent protein (YFP) fluorescence was clearly localized to the plasma membrane, where RIN4, AHA1, and AHA2 have been previously shown to be located. The background fluorescence of chloroplasts in the green channel is due to the excitation at 488 nm. Meanwhile, we were unable to detect any YFP fluorescence between AHA1 or AHA2 and RPS2 (Figure 1B, d, e). As a negative control we co-expressed each protein with the auxin influx carrier AUX1 (At2g38120), an integral plasma membrane protein. None of the proteins were able to induce YFP fluorescence in the presence of the negative control, indicating a specific interaction between AHA1/AHA2 and RIN4 in planta. In order to ensure that the proteins used as negative controls indeed were expressed, expression of AUX1 was detected by western blotting employing the His tag included in the construct (unpublished data) and expression of RPS2 was tested by observation of cell death 48 h after infiltration (unpublished data).

### *RIN4* Overexpression and Knockout Lines Exhibit Differential H^+^-ATPase Activities

**Figure 2:**
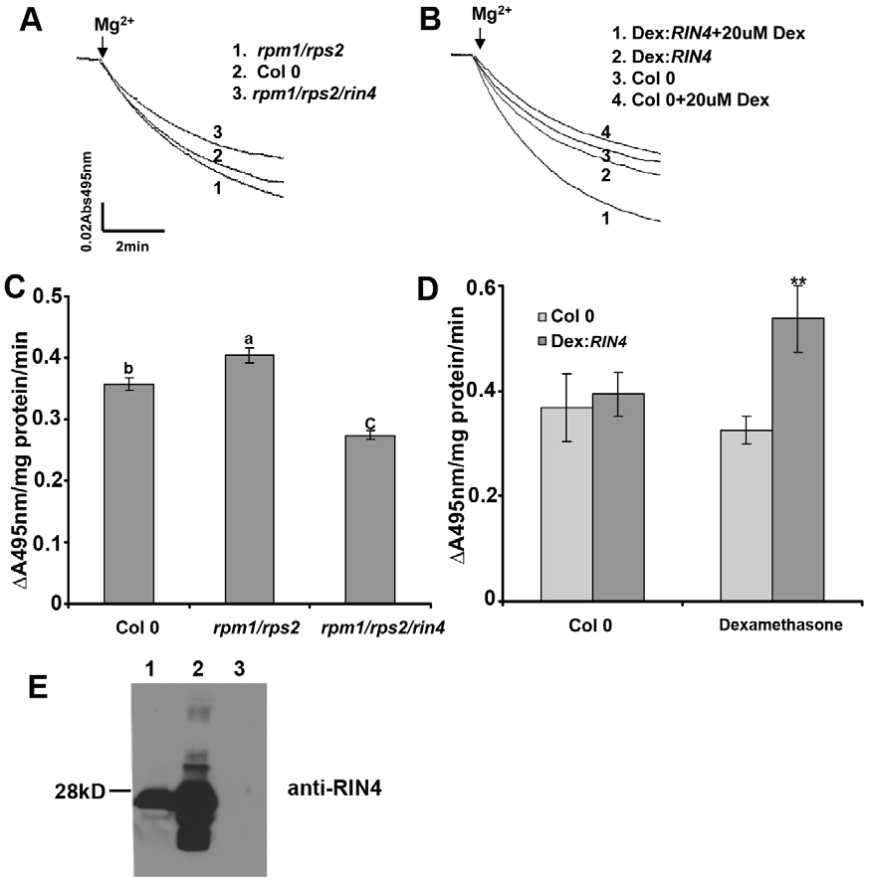
Plasma membrane H^+^-ATPase enzymatic activity is altered in *RIN4* overexpression and knockout lines. Plant leaf plasma membranes were purified by an aqueous polymer two-phase system. The H^+^-pumping activity assay was conducted on inside-out plasma membrane vesicles as described in the Materials and Methods. In this assay, the plasma membrane H^+^-ATPase hydrolyzes ATP and pumps H^+^ into vesicles, which creates the pH gradient across the membrane. The pumping activity was measured by the pH probe acridine orange quenching at an absorbance of 495 nm. (A) and (C) H^+^-pumping activity decreased in the *RIN4* mutant line *rpm1/rps2/rin4*, but not in *rpm1/rps2* plants. (B) and (D) *RIN4* overexpression results in an increase in H^+^-pumping activity in comparison to Col 0. (C) and (D) The initial slope of acridine orange absorbance quenching was graphed from (A) and (B) respectively. H^+^-pumping activity is reported as ΔA_495nm_/mg protein/min. Dexamethasone (Dex) inducible *RIN4* lines and Col 0 were sprayed with water and 0.025% silwett or 20 µM Dex in 0.025% silwett. Leaf tissue was harvested after 48 h, and plasma membranes were immediately purified. (E) RIN4 immunoblot showing RIN4 expression levels in Col 0 (1), Dex:*RIN4* (2), and *rpm1/rps2/rin4* mutant lines (3) 48 h after Dex treatment. Each experiment was repeated two times with independent plasma membrane isolations. Statistical differences were detected by Fisher's LSD [74] alpha = 0.05 for (C) and a two-tailed *t*-test for (D).

RIN4 can interact with the C-terminal regulatory domains of AHA1 and AHA2. Therefore, we investigated the hypothesis that RIN4 can regulate H^+^-ATPase activity. Because it is not possible to measure the biochemical activity of single PM H^+^-ATPase isoforms in planta, we analyzed PM H^+^-ATPase activity as a whole, even though RIN4 may only affect a subset of ATPases. Plasma membrane vesicles were purified from Col 0, dexamethasone (Dex) inducible *RIN4* overexpression [7], *rpm1/rps2*, and *rpm1/rps2/rin4* leaf tissue by aqueous two-phase partitioning. We have used the *rpm1/rps2/rin4* triple mutant for experiments to avoid the weak activation of *RPM1* that occurs in the absence of *RIN4* [37]. PM H^+^-ATPase activity was subsequently measured on inside-out plasma membrane vesicles as described by Palmgren and colleagues [38]. In this assay, the PM H^+^-ATPase hydrolyzes ATP and pumps H^+^ into vesicles, which creates a pH gradient across the membrane. The pumping activity was measured by quenching of the ΔpH probe acridine orange at an absorbance of 495 nm. H^+^ transport measured from plasma membrane vesicles purified from wild-type Col 0 leaves demonstrated that these vesicles were both transport competent and highly enriched for plasma membrane (Figure S3). In *rpm1/rps2/rin4* leaves, H^+^-ATPase activity was 30% lower than Col 0 (*p*<0.001, Figure 2A and 2C). In *RIN4* overexpression lines, H^+^-ATPase activity was 65% higher than Col 0 (Figure 2B and 2D). We also noticed that the *rpm1/rps2* double mutant exhibited slightly higher H^+^-ATPase activity than Col 0 (7%–13%) across independent plasma membrane isolations (*p*<0.05, Figure 2A and 2C). Because both RPS2 and RPM1 interact with RIN4, this line may possess more RIN4 protein that can interact with the H^+^-ATPase, thus increasing its activity. *RIN4* overexpression was induced by spraying the Dex:*RIN4* line with 20 µM Dex and harvesting tissue 48 h later (Figure 2E). We also found that Dex treatment itself slightly inhibited the H^+^-ATPase enzymatic activity in Col 0. This is not surprising, because previous studies have revealed that Dex treatment alone can lead to significant changes in gene expression [39]. Nevertheless, when comparing to Col 0 and Dex:*RIN4* lines after treating with Dex, it is clear that *RIN4* overexpression leads to enhanced PM H^+^-ATPase activity. These results are consistent with the hypothesis that RIN4 can act to regulate H^+^-ATPase activity at the plasma membrane. On the basis of these results, RIN4 acts as a positive regulator of AHA1/AHA2, as *RIN4* overexpression lines exhibit enhanced AHA activity and the *rin4* knockout exhibits decreased AHA activity.

**Figure 3:**
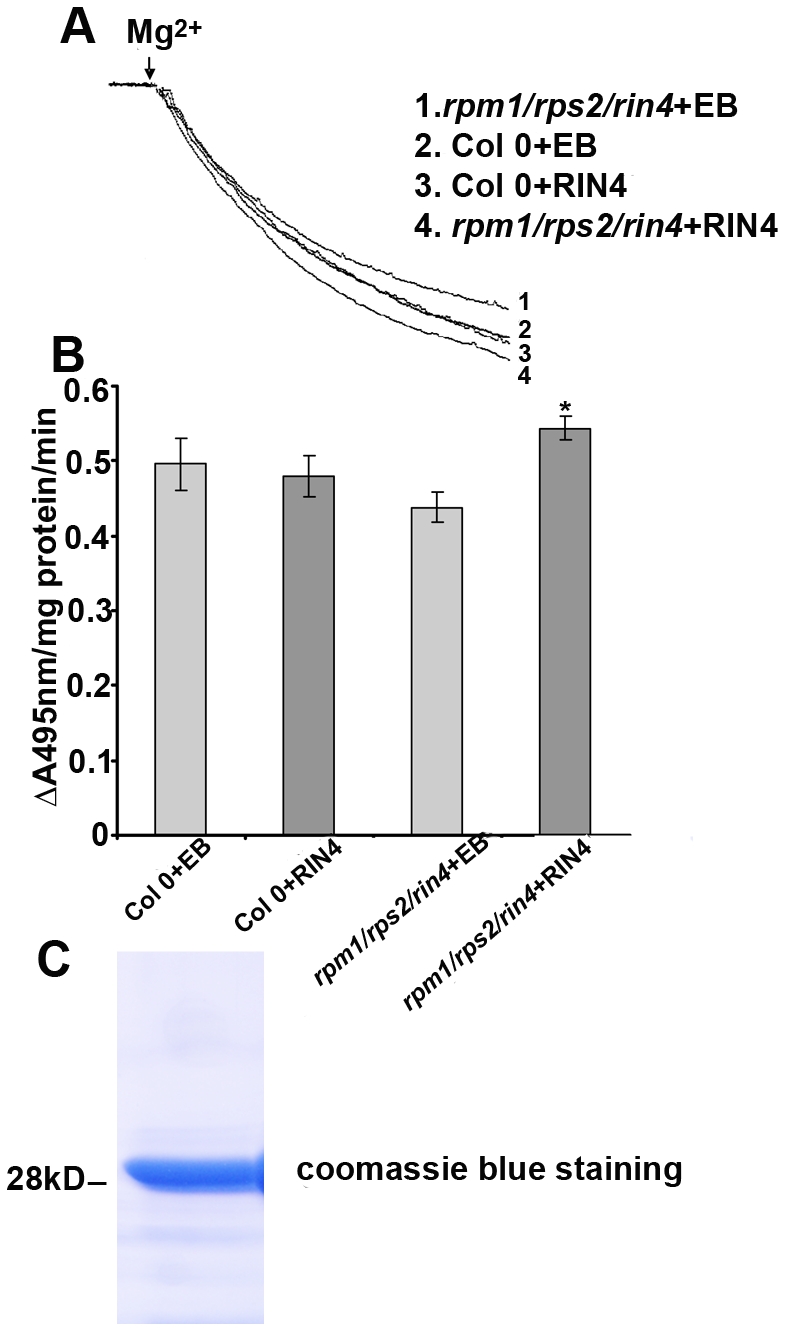
RIN4 positively regulates plasma membrane H^+^-ATPase enzymatic activity in vitro. Purified recombinant RIN4 protein (3 µg) or elution buffer (EB) were added in the assay medium and pre-incubated at 25°C for 10 min. The same activity assay is performed as in Figure 2. (A) RIN4 recombinant protein enhanced H^+^-pumping activity in the *rpm1/rps2/rin4* mutant in vitro, but not in wild-type Col 0. The assays with EB served as the control. (B) The initial slope of acridine orange absorbance quenching was graphed from (A). H^+^-pumping activity is reported as ΔA_495nm_/mg protein/min. (C) An SDS-PAGE gel stained with coomassie blue demonstrating the purity of the recombinant RIN4 protein. Each experiment was repeated two times with independent plasma membrane isolations. Results are shown as the mean (*n* = 3), including standard deviation from plasma membrane vesicles isolated at one time point. Statistical differences were detected with a two-tailed *t*-test.

To test the in vitro effect of RIN4 on H^+^ pumping, recombinant RIN4 protein was purified from *E. coli* and added directly to H^+^ transport assays. H^+^ transport activity in vesicles isolated from the *rpm1/rps2/rin4* knockout was increased in the presence of 3 µg of RIN4 (Figure 3). No effect on H^+^ transport was observed when recombinant RIN4 protein was added to vesicles isolated from wild-type Col 0 plants (Figure 3).

### Constitutively Active PM H^+^-ATPase Mutants Exhibit Enhanced Sensitivity to *Pst* Spray Inoculation

In order to determine if altering the activity of AHA1 or AHA2 could lead to changes in PTI or ETI, we first analyzed *aha1* (salk_118350) and *aha2* (salk_022010) knockout lines. We were unable to detect any obvious morphological or altered disease phenotypes in either knockout line (unpublished data). We were unable to generate an *aha1/aha2* double mutant by crossing salk_118350 and salk_022010, a result that has been reported previously [40]. These results suggest that knocking out both *AHA1* and *AHA2* is a lethal combination, indicating that that *AHA1* and *AHA2* may be functionally redundant in *Arabidopsis*. Therefore, we analyzed *ost2-1D* and *ost2-2D*, which possess point mutations of P_68_S and L_169_F/G_867_S in *AHA1*, respectively, and act as dominant activation mutations [35]. The *ost2-1D* mutant background is in the Landsberg erecta (Ler) ecotype and the *ost2-2D* is in the Col 0 ecotype.

**Figure 4:**
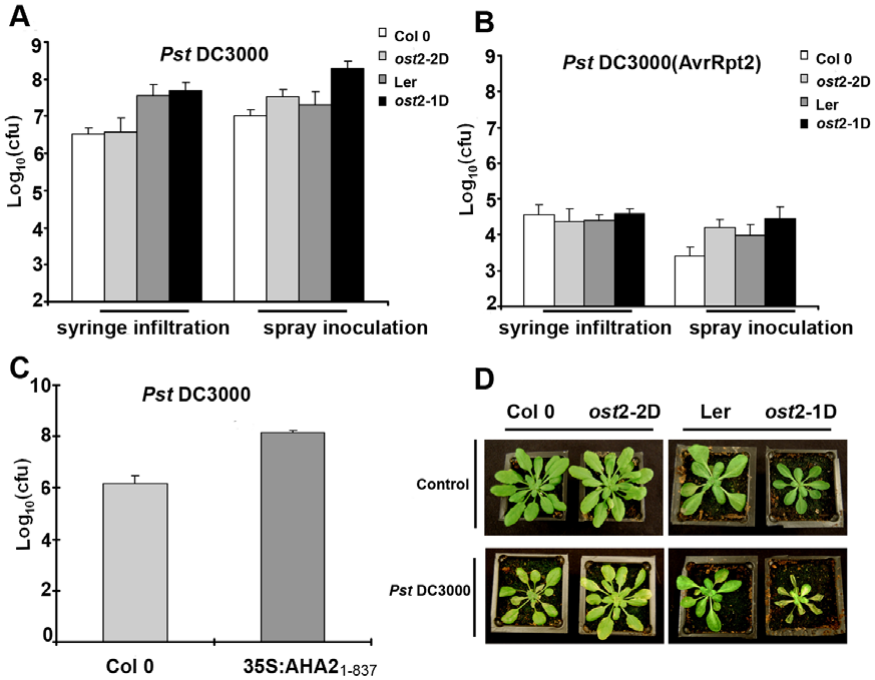
Constitutively active *AHA1* lines display enhanced susceptibility to spray but not syringe inoculation with *P. syringae* pv. *tomato* strain DC3000. (A) 4-wk-old *Arabidopsis* plants were inoculated by syringe infiltration or spray inoculation with *Pst* DC3000 and subjected to growth curve analysis 3 d post-inoculation. (B) *Arabidopsis* plants were inoculated by syringe infiltration or spray inoculation with *Pst* DC3000 (AvrRpt2) and subjected to growth curve analysis 3 d post-inoculation. (C) The 35S:*AHA2_(1–837)_* overexpression line displays enhanced susceptibility to spray inoculation with *Pst* DC3000. (D) Disease symptoms in *ost2-2D*, *ost2-1D*, Col 0, and Landsberg (Ler) 3 d after spray inoculation with *Pst* DC3000 (bottom panel). Experimental control plants were sprayed with water (top panel). All syringe inoculations were performed at a concentration of 0.5×10^5^ CFU/ml; spray inoculations were performed at a concentration of 1×10^9^ CFU/ml. Results are shown as the mean (*n* = 6), including standard deviation.

The *ost2-1D* and *ost2-2D* mutants were originally identified based on their open stomata phenotype [35]. Because stomata can serve as ports of entry for microbial pathogens, we hypothesized that these mutants may facilitate enhanced bacterial entry inside leaves. We were unable to detect a difference between Col 0, Ler, and *ost2-1D* or *ost2-2D* after syringe infiltration with virulent *Pst* DC3000 or avirulent *Pst* DC3000 expressing the effector AvrRpt2, which induces ETI (Figure 4A and 4B). Col 0 and Ler exhibited clear bacterial speck symptoms by 4–5 d after spray inoculation. However, the leaves of *ost2-2D* lines were completely collapsed by 4 d after spray inoculation. Therefore, all growth curves were performed at 3 d post-inoculation, when disease symptoms were clearly visible on *ost2-1D* and *ost2-2D* (Figure 4D). When we spray-inoculated with *Pst* DC3000 or *Pst* DC3000 (AvrRpt2), the bacteria were able to grow 5- to 10-fold more in the *ost2-1D* and *ost2-2D* mutant lines compared to Ler and Col 0 and displayed enhanced disease symptoms (Figure 4A, 4B, and 4D). These results show that AHA1 activation can facilitate *Pst* entry into the plant leaf interior.

Our genetic analysis suggests that *AHA1* and *AHA2* are functionally redundant. Therefore, we hypothesized that *AHA2* overexpression lines would also enable enhanced bacterial entry into the leaf interior. AHA2 regulation has been well-studied in vitro, and the C terminus acts as a negative regulator of the PM H^+^-ATPase [36],[41],[42]. Removing the C terminus induces strong auto-activation in vitro and in planta [41],[43]. We generated an *AHA2* overexpression line in Col 0 by transforming a truncated version of *AHA2* (amino acids 1–837) without its C-terminal inhibitory domain under the control of the cauliflower mosaic virus 35S promoter. Because of the small leaf size of the 35S:*AHA2*_1–837_ line, we were unable to syringe inoculate or harvest large quantities of leaf tissue necessary for PM H^+^-ATPase enzymatic analysis. The resulting transgenic plants were dwarf with pronounced leaf chlorosis, decreased germination rates, and possessed enhanced AHA2 expression (Figure S4). *Pst* DC3000 was able to grow 20-fold more in this line compared to Col 0 after spray inoculation (Figure 4C). However, the 35S:*AHA2*_1–837_ line did not have a constitutively open stomata phenotype like *ost2-1D* and *ost2-2D* mutants (unpublished data). The pleotropic phenotypes generated by overexpressing *AHA2*_1–837_ in Col 0 are not surprising because strong constitutive activation of plasma membrane H^+^-ATPase(s) can result in a nonspecific expression in different cell types, profound changes in plasma membrane potential, and will affect multiple biological processes [43]. For these reasons, we did not investigate the 35S:*AHA2*_1–837_ line further and concentrated our analyses on the *AHA1* activation mutants.

The *ost2-1D* and *ost2-2D* mutants were previously reported as lesion-mimic mutants and displayed salicylic acid-induced necrosis on leaflets [35]. Under standard growth conditions for pathogen inoculation, we did not observe this phenotype on any of the lines exhibiting enhanced PM H^+^-ATPase activity (Figure 4D). However, we were able to visualize leaflet necrosis on both lines when they were grown under conditions to promote flowering (140 µmol/sec/m^2^, 16-h days, 23°C). The phenotypes of lesion-mimic mutants can be variable and sensitive to variations in growth conditions [44]. Lesion-mimic mutants are often associated with mutations in ion channels [44],[45]. As the *AHA* family is an important regulator of multiple cellular processes, spatial and temporal regulation of PM H^+^-ATPases inside mesophyll cells may also be an important component of plant immune signaling.

**Figure 5:**
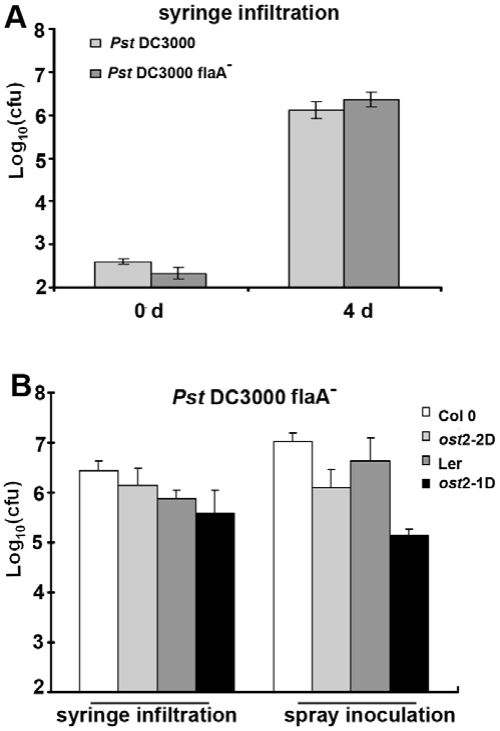
Constitutively active *AHA1* lines do not exhibit enhanced susceptibility to nonmotile *P. syringae* pv. *tomato.* (A) Col 0 plants were inoculated by syringe infiltration with 0.5×10^5^ CFU/ml *Pst* DC3000 and the nonmotile flagellin mutant *Pst* DC3000 *flaA* ^−^. Bacterial growth was measured 3 d post-inoculation. (B) Constitutively active *AHA1* mutants and corresponding wild-type *Arabidopsis* ecotypes were inoculated by syringe infiltration and spray inoculation with *Pst* DC3000 *flaA* ^−^ and subjected to growth curve analysis 3 d post-inoculation. All syringe inoculations were performed at a concentration of 0.5×10^5^ CFU/ml; spray inoculations were performed at a concentration of 1×10^9^ CFU/ml. Results are shown as the mean (*n* = 6), including standard deviation.

In order to test the hypothesis that enhanced bacterial growth on *ost2* mutant leaves is due to their increased ability to gain entry into the leaf interior via open stomata, we inoculated wild-type *Arabidopsis* and *ost2* mutant lines with the nonmotile *Pst* flagellin mutant *flaA* [46]. The *flaA* mutant grew to similar levels as wild-type *Pst* when syringe infiltrated in Col 0 leaves (Figure 5A). We were unable to detect enhanced growth of the *flaA* mutant after spray inoculation onto *ost2-1D* and *ost2-2D*, indicating that these mutant plants promote bacterial colonization of the leaf by allowing bacteria to gain entry by swimming through their stomatal apertures (Figure 5B). Interestingly, we noticed that growth of the *flaA* mutant was decreased in *ost2* mutants after spray inoculation, but not syringe infiltration (Figure 5B). This may be due to an inability of the *flaA* mutant to swim away from unfavorable microenvironments (such as low pH) near stomatal openings with enhanced PM H^+^-ATPase activity.

### The Stomata of rin4 Mutant Plants Cannot Be Re-Opened by Virulent Pst DC3000

**Figure 6:**
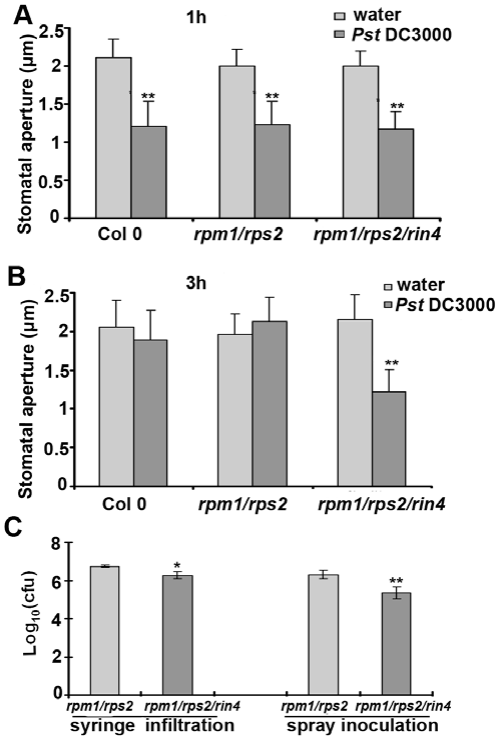
*rin4* knockout lines do not re-open their stomata after exposure to virulent *Pst* DC3000. *Pst* DC3000 induces stomatal closure at 1 h (A) and re-opening after 3 h (B) on Col 0 but not *rin4* knockout lines. (C) The *rpm1/rps2/rin4* mutant displays enhanced resistance to spray inoculation with *Pst* DC3000. 4-wk-old *rpm1/rps2* and *rpm1/rps2/rin4* plants were syringe infiltrated or spray inoculated with *Pst* DC3000 and leaves were subjected to growth curve analyses 4 d post-inoculation. Bacterial growth curve results are shown as the mean (*n* = 6), including standard deviation. These experiments are representative of at least three independent replicates. In this and all other figures, results are shown as the mean (*n* = 50–80) stomata ±SEM and statistical differences were detected with a two-tailed *t*-test.

Lines exhibiting increased AHA1 activity are more susceptible to bacterial inoculation due to their open stomata phenotype (Figures 4 and 5). Previously, Melotto and colleagues showed that upon perception of PAMPs, Col 0 stomata will close within 1 h [15]. Virulent *Pst* can re-open stomata after 3 h through the production of coronatine, facilitating pathogen entry. Because RIN4 can interact with the C-terminal regulatory domain of AHA1 and AHA2 (Figure 1), we investigated the stomatal response in the *rin4* knockout line after pathogen inoculation. Leaf epidermal peels from Col 0, *rpm1/rps2*, and *rpm1/rps2/rin4* were floated on 1×10^8^ colony-forming unit (CFU)/ml *Pst* DC3000 and their stomatal apertures were measured in response to pathogen inoculation. Stomatal apertures from all genotypes closed after 1 h (Figure 6A). Importantly, we observed that *Pst* DC3000 could not re-open the stomata in *rpm1/rps2/rin4* after 3h (Figure 6B). The stomata of *rpm1/rps2* lines were open after 3 h, indicating that this phenotype is solely due to the lack of *RIN4* (Figure 6B). We also tested *ndr1-1* mutant plants for a defect in stomatal response to PAMPs, but *ndr1-1* lines were still able to re-open their stomata 3 h after exposure to *Pst* DC3000 (unpublished data), indicating that *NDR1* is not required for the *RIN4-*mediated stomatal phenotype. These observations are consistent with *RIN4* being a negative regulator of plant innate immunity. These results also support the hypothesis that RIN4 and AHA1/AHA2 work together to regulate stomatal apertures in response to PTI.

Previously, the *rpm1/rps2/rin4* triple mutant was shown to be more resistant than *rpm1/rps2* after spray inoculation with *Pst* DC3000 [7]. In addition, *rin4* knockout lines exhibit enhanced callose deposition in response to PTI, whereas *RIN4* overexpression lines display the opposite phenotype [7]. Therefore, *RIN4* may play a role in PTI signaling in both guard cells and mesophyll cells. In order to test this hypothesis, we inoculated *rpm1/rps2* and *rpm1/rps2/rin4* plants grown under the same conditions by both spray inoculation and syringe infiltration. Spray inoculation always resulted in a significant decrease of 4- to 9-fold in bacterial growth on the *rpm1/rps2/rin4* mutant when compared to *rpm1/rps2* (Figure 6C). We were also able to detect a slight decrease (2- to 4-fold) in bacterial growth on the *rpm1/rps2/rin4* mutant after syringe infiltration. These results indicate that *RIN4* contributes significantly to PTI signaling in guard cells and has a subtle phenotype with respect to PTI in mesophyll cells. Because *rin4* knockout lines do not re-open their stomata after inoculation with *Pst*, this may be the reason why lines lacking *RIN4* exhibit increased resistance to virulent bacteria after spray inoculation.

### *RIN4* Is Expressed in Both Guard Cells and Mesophyll Cells

**Figure 7:**
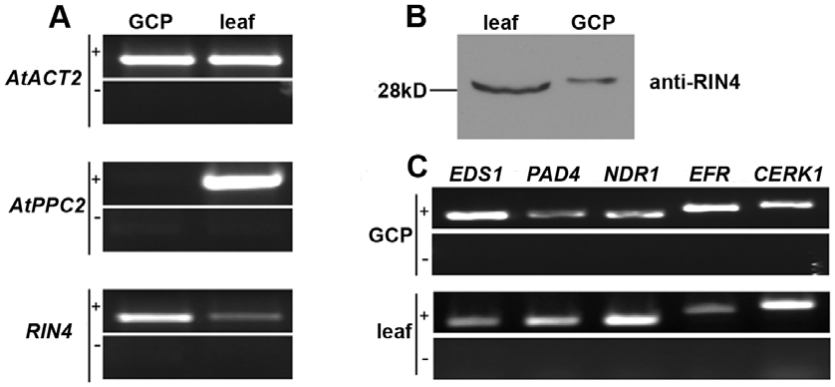
*RIN4* is expressed in guard cells. Guard cell protoplasts (GCPs) were purified from Col 0 leaves and visually inspected for purity by light microscopy. Half of the GCP sample was used for RNA extraction and half for total protein extraction. (A) RNA was isolated from entire Arabidopsis leaves or GCPs and subjected to RT-PCR. *RIN4* mRNA is highly expressed in guard cells. The expression of phosphoenolpyruvate carboxylase 2 (*ATPPC2*, At2g42600), which has low-level expression in guard cells and high-level expression in mesophyll cells served a control for guard cell protoplast purity [47]. Actin (*AtACT2*) served as a loading control. (B) Anti-RIN4 immunoblots detected RIN4 protein expression in both Col 0 leaf tissue and GCPs. Thirty µg of total protein extract was loaded per lane. (C) RNA samples from (A) were subjected to RT-PCR to detect the expression of additional innate immune signaling components. *EDS1*, *PAD4*, *RPS2*, *NDR1*, *EFR*, and *CERK*1 transcript levels were detected in GCPs and leaf tissue after a 28-cycle amplification. +, RT-PCR, −, no-RT control.

Our observation that virulent *Pst* cannot re-open stomata in *rin4* knockout lines led us to investigate what cell types express *RIN4*. We investigated *RIN4*'s expression pattern in intact leaves and guard cells. Guard cell protoplasts were isolated from Col 0, visually inspected for purity, and analyzed for the presence of *RIN4* (Figure 7A). We used the expression of phosphoenolpyruvate carboxylase 2 (*ATPPC2*, At2g42600), which has low-level expression in guard cells and high-level expression in mesophyll cells, as a control to verify guard cell protoplast purity [47]. Each batch of purified guard cell protoplasts was divided in two for extraction of RNA and protein. Our reverse transcriptase (RT)-PCR analysis showed that *RIN4* was expressed in both Col 0 guard cells as well as intact leaves (Figure 7A). Guard cells make up less than 2% of the leaf epidermal cells, which highlights the expression of *RIN4* within guard cells. Next, we performed immunoblot analysis on leaf and guard cell protoplast protein extracts (30 µg) with the anti-RIN4 antibody. RIN4 protein was detected in Col 0 guard cells as well as in the intact leaf (Figure 7B). Given the abundance of mesophyll cells in the leaf sample, this result indicates that RIN4 is strongly expressed in guard cells.

*AHA1* expression in guard cells was previously demonstrated [35]. On the basis of our interaction studies we therefore tested if *AHA2* is also expressed in guard cells. Transgenic plants expressing an *AHA2* promoter:*GUS* construct clearly demonstrated AHA2 expression in guard cells (Figure S4C) supporting the hypothesis that both AHA1 and AHA2 interact with RIN4 and that this interaction is physiologically relevant.

Given the importance of guard cells in regulating bacterial invasion, we investigated if additional immune signaling components were present in guard cells. Like Melotto and colleagues [15], we were able to detect the flagellin PAMP receptor *FLS2* (unpublished data). We detected expression of the EF-Tu PAMP receptor *EFR* and the chitin PAMP receptor *CERK1* in Col 0 guard cell protoplasts by RT-PCR (Figure 7C). We were also able to detect the expression of *EDS1*, *PAD4*, and *NDR1*, which are involved in the manifestation of ETI (Figure 7C). By mining publicly available microarray data from Yang and colleagues [48], we analyzed the expression of the following genes in both guard cell and mesophyll cell protoplasts: *FLS2*, *EFR*, *CERK1*, *EDS1*, *PAD4*, *NDR1*, *RPS2*, *RPM1*, and *RIN4*. With the exception of *CERK1*, all genes were expressed at a detectable level in both guard cells and mesophyll cells (unpublished data).

### The Stomata of *ost2-1D* and *ost2-2D* Do Not Respond to PTI-Mediated Stomatal Closure

**Figure 8:**
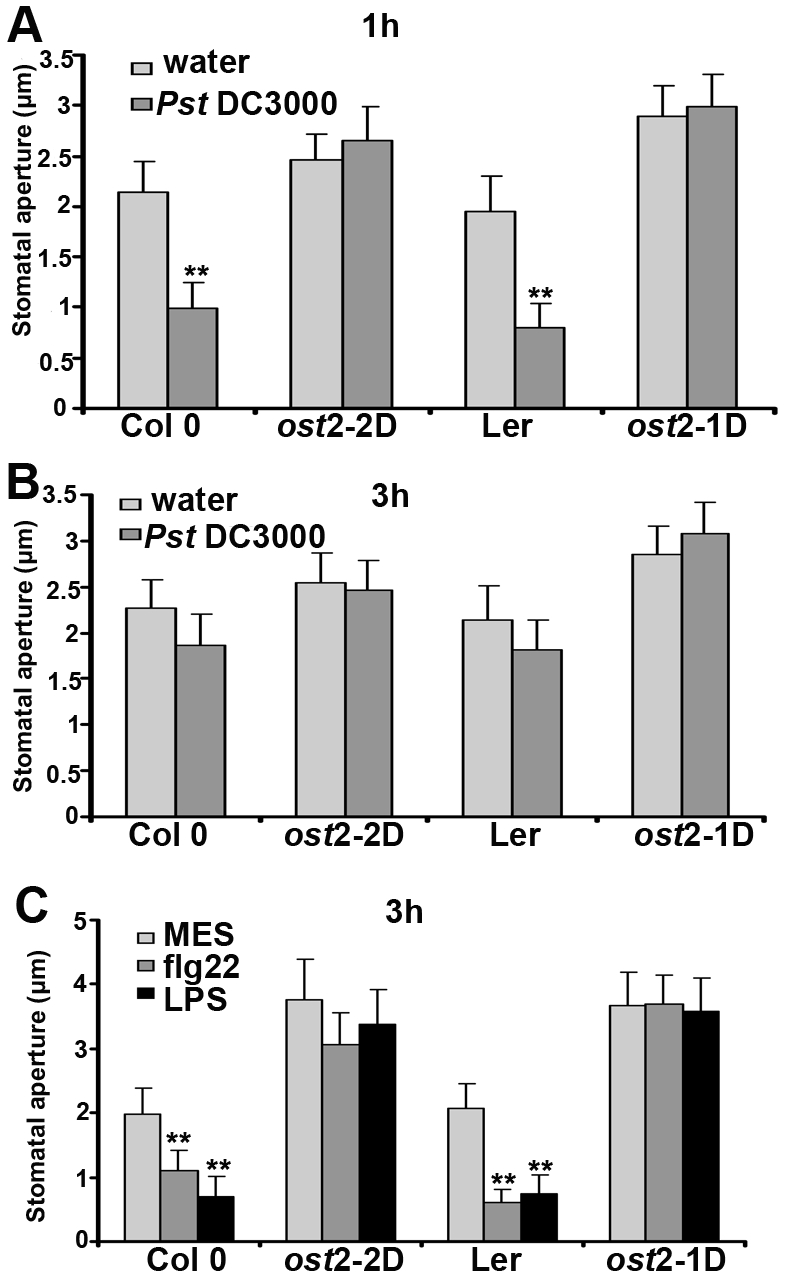
*AHA1* constitutively active mutant lines are insensitive to PTI-mediated stomatal closure. Stomatal apertures were measured in epidermal peels of wild-type Col 0 and Ler, as well as the *AHA1* mutants *ost2-2D* and *ost2-1D* after incubation with water or 1×10^8^ CFU/ml *Pst* DC3000 for 1 h (A) and 3 h (B). (C) *Arabidopsis* epidermal peels were floated on MES buffer containing the flagellin peptide Flg22 (5 µmol/l) and LPS 100 ng/µl). Stomatal apertures were measured after 3h. MES served as a control.

The stomata of *ost2* mutants are ABA insensitive, but do respond to other stimuli such as CO_2_ and blue light, indicating that individual PM H^+^-ATPases may exhibit defined biological roles [35]. Therefore, we investigated the ability of *ost2* mutant lines to respond to PTI-mediated stomatal closure. We floated epidermal peels of Ler, Col 0, and *ost2* mutant lines on 1×10^8^ CFU/ml *Pst* DC3000 and measured their stomatal apertures in response to pathogen inoculation. *Pst* could not induce stomatal closure in *ost2-1D* or *ost2-2D,* while 80% of the stomata from Col 0 and Ler were closed after 1 h (Figure 8A, 8B). Epidermal peels from *ost2* mutants were also incubated with the flg22 peptide of flagellin and lipopolysaccharide (LPS), which are recognized as bacterial PAMPs. We clearly observed that incubation with 10 nM/ml flg22 or 100 µM LPS can induce stomatal closure in either Ler or Col 0 plants, but not in *ost2-1D* or *ost2-2D* (Figure 8C), suggesting that AHA1 inactivation contributes to stomatal closure during PTI signaling.

PTI induces an oxidative burst within minutes after pathogen perception, and treatment with reactive oxygen species, such as H_2_O_2_ and nitric oxide (NO) results in stomatal closure [49]. We were interested in determining if stomata from plants with enhanced AHA1 activity would respond to the presence of reactive oxygen species. In Figure S5, we treated plants with 0.2 mM H_2_O_2_ and 100 µM sodium nitroprusside (SNP, an NO donor). Neither H_2_O_2_ nor SNP could induce closure in *ost2-1D* and *ost2-2D*, but could rapidly induce stomatal closure in wild-type *Arabidopsis*. These results demonstrate that the stomata of *ost2* mutants, which exhibit enhanced AHA1 activity, do not close in response to PTI, therefore enabling virulent bacteria to gain entry into the plant apoplast. Melotto and colleagues also demonstrated that PAMP-induced stomatal closure required the *OST1* protein kinase, a key component of the ABA signaling pathway [15].

## Discussion

**Figure 9:**
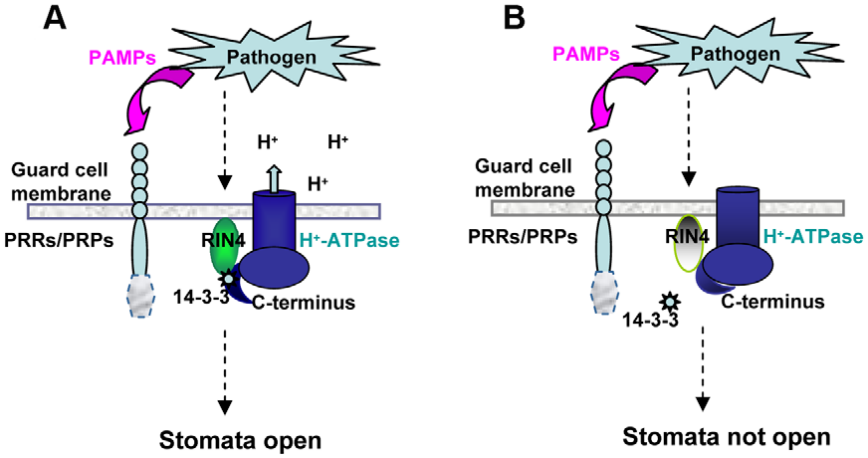
Model of PAMP-induced stomatal closure. RIN4 acts in concert with AHA1 and/or AHA2 to regulate stomatal apertures in response to pathogen attack during PTI. (A) Virulent pathogens are able to overcome PTI and induce stomata to re-open 3 h after pathogen perception. Activation of the PM H^+^-ATPase can lead to hyperpolarization of the plasma membrane and subsequent induction of inward K^+^ channels. These events lead to an increase in guard cell turgor and stomatal opening. (B) RIN4 is a negative regulator of plant innate immunity. In resistant genotypes, pathogens are not able to overcome PTI and stomata remain closed after pathogen perception. Pathogen PAMPs are detected by pattern recognition receptors (PRRs) and the induction of PTI induces stomatal closure. Posttranslational modification of RIN4 (elimination or possibly phosphorylation) inhibits the association between RIN4 and AHA1/AHA2, resulting in inactivation of the PM H^+^-ATPase.

Recognition of pathogens by the host innate immune system is a critical component controlling survival and fitness of both animals and plants. We investigated the function of RIN4, an *Arabidopsis* protein that acts as a negative regulator of both PTI and ETI [7],[8],[11],[13]. Here, we have identified six novel RIN4 associated proteins. We have investigated the association between RIN4 and PM H^+^-ATPases AHA1 and AHA2 in detail. These data are consistent with the model of RIN4 acting in concert with the PM H^+^-ATPases AHA1 and AHA2 to regulate stomatal apertures in response to pathogen attack in resistant genotypes (Figure 9).

Stomata are surrounded by a pair of two guard cells, whose turgor controls opening and closure of the aperture. Changes in the turgor of guard cells are strongly influenced by the activity of PM H^+^-ATPase. Activation of PM H^+^-ATPase can lead to hyperpolarization of the plasma membrane and subsequent induction of inward K^+^ channels resulting in an increase in turgor due to concomitant entry of water and stomatal opening. In contrast, inhibiting the PM H^+^-ATPase and anion channel activation initiate plasma membrane depolarization, resulting in the activation of outward rectifying K^+^ channels [50],[51]. These ion effluxes result in a loss of guard cell turgor and stomatal closure. A number of secondary messengers are important for initiating membrane depolarization, including reactive oxygen species and Ca^2+^.

We have demonstrated that the RIN4 protein acts in concert with PM H^+^-ATPases to regulate stomatal apertures during PTI. Importantly, the *rin4* knockout line does not re-open its stomata in response to virulent *Pst* (Figure 6). This result solidifies the importance of RIN4 in regulating stomatal apertures in response to pathogen attack. Previously, RIN4 was found to be a negative regulator of both PTI and ETI [7],[8],[11],[13]. Our results were consistent with these findings and suggest that RIN4's association with AHA1 and AHA2 is an important component of RIN4 function. Autoactive *AHA1* mutants display increased susceptibility to virulent *Pst*, because of the bacteria's enhanced ability to gain access to the plant interior via open stomata (Figures 4 and 5). *RIN4* overexpression lines exhibit enhanced disease susceptibility and increased PM H^+^-ATPase activity. Conversely, *rin4* knockout lines exhibit decreased disease susceptibility and lower PM H^+^-ATPase activity (Figures 2 and 6C). These results can now explain how RIN4 acts to regulate plant innate immunity at the level of pathogen invasion.

Despite the importance of *RIN4* in plant innate immunity, the pattern of *RIN4* expression remained unknown. Using a combination of RT-PCR, western blotting, and microarray analyses we were able to demonstrate that *RIN4* is expressed in guard cells (Figure 7). These results highlight the importance of *RIN4* in PTI-induced stomatal closure. We were also able to detect the expression of multiple PAMP receptors, R genes, and innate immune signaling components in guard cells at the RNA level, emphasizing the importance of this cell type in the innate immune response (Figure 7).

Inhibition of the PM H^+^-ATPase is one of the first steps required to induce stomatal closure. These data are consistent with a model in which RIN4 acts in concert with AHA1 and/or AHA2 to regulate stomatal apertures in response to pathogen attack during PTI (Figure 9). Perception of the flagellin flg22 peptide during PTI was found to inhibit both inward and outward rectifying K^+^ channels [52]. Therefore, flagellin perception can not only induce stomatal closure, but can inhibit stomatal opening [15],[52]. Because stomata serve as points of entry for multiple bacterial, fungal, and oomycete pathogens, it is not surprising that several different classes of pathogens have evolved to manipulate stomatal apertures during pathogenesis. For example, the polyketide toxin coronatine, produced by several strains of *P. syringae*, can induce stomatal opening after PTI-mediated closure [15]. Coronatine can reverse the inhibition of inward rectifying K^+^ channels, leading to stomatal opening [52]. *Xanthomonas campestris* employs a small diffusible signal molecule, which can also induce stomatal opening on compatible hosts [19]. The most well-characterized example of stomatal manipulation by a pathogen is the toxin fusicoccin, produced by the fungal pathogen *F. amygdali*, the causal agent of almond and peach canker [24]. Fusicoccin is a potent activator of the PM H^+^-ATPase and strongly induces stomatal opening by binding to and stabilizing an activated H^+^-ATPase/14-3-3 complex [25],[27],[53]. These studies highlight the importance of stomatal regulation during plant innate immunity, as components of the signaling pathways controlling stomatal apertures can be regulated by the plant immune system as well as by virulent pathogens.

What is the mechanism RIN4 uses to regulate PM H^+^-ATPase activity? PM H^+^-ATPase regulation has been well studied over the last 20 years (reviewed in [54]). Both crystallographic data and homology modeling of the PM H^+^-ATPase indicate that it possesses a similar structure to other P-type ATPases [55],[56]. The PM H^+^-ATPase also possesses an extended C terminus [57], which is lacking in other P-type ATPases [57] and is involved in negative regulation of pump activity [58]. Activation of the PM H^+^-ATPase can be achieved by phosphorylation of the penultimate threonine residue. Phosphorylation of this residue leads to subsequent binding of regulatory 14-3-3 proteins, which displace the autoinhibitory C-terminal domain. This apparently induces the formation of a dodecamer consisting of six H^+^-ATPase and six 14-3-3 molecules in the PMA2 H^+^-ATPase isoform from *N. plumbaginifolia* [54],[56]. Additional phosphorylated residues have recently been identified that can contribute to both positive and negative regulation of the PM H^+^-ATPase, highlighting the complexity of this pump's regulation [59]–[61].

Data presented in this manuscript are consistent with RIN4 being a positive regulator of the PM H^+^-ATPases AHA1 and AHA2. Previous studies have demonstrated that RIN4 is phosphorylated in planta [8],[62]. It will be interesting to test if the phosphorylation status of RIN4 plays a role in regulating PM H^+^-ATPase activity. Future research investigating if RIN4 is transcriptionally or posttranslationally modulated during the guard cell response to PAMPs and *Pst* DC3000 may help elucidate the mechanism employed by RIN4 to regulate the PM H^+^-ATPase. In addition, *RIN4* homologs can be detected in many plants where substantial DNA sequences are available. In the future, it will be important to determine the role of *RIN4* as well as RIN4-associated proteins across different species. For example, stomatal closure in response to PTI occurs in multiple plants [15],[18]. Does the association of RIN4 with PM H^+^-ATPases act to regulate stomatal apertures in other species?

It will also be important to elucidate how innate immune complexes change in response to pathogen attack and if complex constituents are the same between different cell types. It is plausible that components of the innate immune complexes exist in distinct pools within each cell, with each pool controlling different aspects of PTI and ETI. There is evidence for RIN4 existing in different cellular pools within plant leaves based on data obtained from co-immunoprecipitation experiments [8],[11],[13]. In this study, we were able to elucidate members of the RIN4 complex in the absence of pathogen infection. An in-depth investigation how the RIN4 complex assembles and changes during PTI, ETI, and after pathogen-induced modification in different cell types (e.g., guard cells and mesophyll cells) and plant genotypes will greatly facilitate our understanding of innate immune signaling.

## Materials and Methods

### Plant Materials

*Arabidopsis* plants, Columbia (Col 0), Landsberg erecta (Ler), and the mutants derived from them as indicated in the figures were sown in soil and stratified at 4°C for 2 d. In the text, the *rps2*, *rpm1*, and *rin4* mutants refer to *rps2-101c*, *rpm1-3*, and the *rin4* T-DNA knockout [8],[9],[63]. Dex:*RIN4* lines were previously described, and all figures refer to line 31 [7]. Plants were grown in controlled environment chamber at 24°C with a 10-h light/14-h dark photoperiod under a light intensity of 85 µE/m^2^/s. For all the experiments, 4–5 wk old plants were used. 35S:*AHA2*_(1–837)_ transgenic lines were generated by following the standard floral dip transformation procedure [64]. The *AHA2* (1–837) fragment was cloned into the BamH I/Xho I site of binary vector pMD-1 and transgenic plants were screened on 50 µg/ml kanamycin. Two independent T3 lines were used for bacterial inoculation.

### Bacterial Strains and Inoculations

*Pst* DC3000, *Pst* DC3000 (AvrRpt2), and the flagellin deficient mutant *Pst* DC000 *flaA* ^−^ were grown on NYG plates for 30 h, then cultured at 28°C in NYG media for 48 h [46]. *Pst* DC3000 (AvrRpt2) expressed AvrRpt2 from the broad-host range vector pDSK519 [65]. Antibiotics were used for plate selection at the following concentrations: 25 µg/ml kanamycin, 100 µg/ml rifampicin, and 35 µg/ml chloramphenicol. For spray inoculation, *Arabidopsis* leaves were sprayed until runoff with a Preval sprayer containing 1×10^9^ CFU/ml bacteria in 10 mM MgCl_2_ with 0.025% silwett L-77. Inoculated plants were left uncovered for 30 min and then covered with a plastic dome for 2 d. For syringe infiltration, bacteria were resuspended in 10 mM MgCl_2_ and inoculated at a concentration of 0.5×10^5^ CFU/ml with a needleless syringe. Leaves were surface sterilized for 30 s in 70% ethanol, and bacterial populations were determined by growth curve analysis as described by Kim and colleagues [7]. All experiments were repeated at least three times, with a minimum of three biological replicates per time point.

### Stomatal Aperture Measurements

Stomatal aperture measurements were conducted according to a published procedure [15]. Plants were induced to open stomata under white light for 2 h. Epidermal peels were floated on a 1×10^8^ CFU/ml of *Pst* in water or purified PAMPs. For PAMP treatments, epidermal peels were floated on 5 µM flg22 peptide (synthesized by GenScript) in MES buffer (10 mM KCl, 0.2 mM CaCl_2_, 10 mM MES-KOH [pH 6.15]), 100 ng/µl LPS (Sigma) in MES buffer or MES buffer alone as a negative control. Stomatal apertures were analyzed by microscopy with a digital camera and measured with SPOT4.1 software (Diagnostic Instruments) at 0-h, 1-h, and 3-h timepoints. All experiments were repeated at least three times, with a minimum of three biological replicates per time point.

### Plasma Membrane H^+^-ATPase Activity Assays

*Arabidopsis* plants were grown as described above for 5 wk in soil at a pH of 7.5. To determine the effect of overexpressing *RIN4*, Dex:*RIN4* and Col 0 leaves were sprayed with water and 0.025% silwett or 20 µM Dex in 0.025% silwett. Leaf tissue was harvested after 48 h. For all experiments, plasma membranes were immediately purified after harvesting leaf tissue. *Arabidopsis* leaves (30 g) were homogenized with a blender in 200 ml ice-cold buffer containing 50 mM MOPS (pH 7.0), 0.33M sucrose, 5 mM EDTA, 2 mM DTT, 1.5 mM ascorbate, 0.2% (w/v) insoluble polyvinylpolypyrrolidone, 1 mM phenylmethylsulfonyl fluoride, 1 µg/ml leupeptin, and 1 µg/ml pepstain A. Plasma membranes were purified from the microsomal fraction (10,000 g to 50,000 g pellet) by partitioning at 4°C in an aqueous polymer two-phase system as described previously [66]. The final plasma membrane pellet was suspended in re-suspension buffer (5 mM potassium phosphate buffer [pH 7.8], 0.33 M sucrose, 10% (v/v) glycerol, 50 mM KCl, 0.1 mM EDTA, 2 mM DTT, 1 µg/ml leupeptin, and 1 µg/ml pepstain A). H^+^-pumping activity was detected by a decrease of acridine orange absorbance at 495 nm [38]. The assay buffer contained 20 mM MES-KOH (pH 7.0), 140 mM KCl, 3 mM ATPNa_2_, 30 µM acridine orange, 0.05% Brij 58, and 50 µg of plasma membrane protein in a total volume of 1 ml. Membranes were pre-incubated at 25°C for 5 min in assay buffer. The assay was initiated by the addition of 3 mM MgSO_4_. To determine if purified RIN4 protein could alter H^+^-pumping activity in vitro, 3 µg of purified recombinant RIN4 protein was added to the assay medium and pre-incubated at 25°C for 10 min before the addition of MgSO_4_. Recombinant RIN4 protein was expressed in *E. coli* and purified by Ni^+^ affinity chromatography as described previously [14].

The Bradford assay was used to calculate total plasma membrane protein content [67]. Each experiment was repeated two times with independent plasma membrane isolations.

### Yeast Two-Hybrid

The yeast strain AH109, containing the HIS3 and lacZ reporter genes, was used for yeast two-hybrid analyses (Matchmaker, Clontech). The coding sequence of *RIN4*, *AHA1*_(837–950)_, and *AHA2*_(837–949)_ fragments were obtained by PCR amplification and sequenced. The RIN4 PCR product was cleaved and cloned into the BamH I/Pst I site of the pGBKT7 vector (binding domain). *AHA1*_(837–950)_ and *AHA2*_(837–949)_ PCR products were cloned into the EcoR I/Xho I sites of pGADT7 vector (activation domain). pGBKT7-*RIN4*, pGADT7-*AHA1*_(837–950)_, pGADT7-*AHA2*_(837–949)_, the positive control pGAL4 and the negative control pGBKT7 vector were all transformed into the yeast strain AH109 following the manufacturer's protocol. Protein expression was detected in transformed strains by immunoblotting. Transformants were dilution plated onto yeast potato dextrose agar (YPDA) and synthetic dextrose lacking leucine/tryptophan/histidine (SD-3). Yeast growth was examined as previously described [28].

### Bimolecular Fluorescence Complementation

#### Constructs used for BiFC experiments

AHA1, AHA2, RIN4, RPS2, and AUX1 were amplified by PCR. The PCR products included no stop codons and a CACC overhang in the 5′ end for directional cloning into the pENTR/D-TOPO vector. The entry clones were sequenced and cloned into gateway compatible BiFC vectors by LR reactions. RIN4 was made with an N-terminal fusion resulting in cCFP-RIN4 (pMP2869) and nYFP-RIN4 (pMP2870). RPS2, AHA1, AHA2, and AUX1 were made with C-terminal fusions resulting in the following constructs: RPS2-nYFP (pMP2872), AHA1-nYFP (pMP2317), AHA2-cCFP (pMP2256), AHA2-nYFP (pMP2457), AUX1-cCFP (pMP2879).

#### Transient expression of BiFC constructs in *N. benthamiana*

For introduction of constructs into *N. benthamiana* leaves, *Agrobacterium* strain C58C1 was transformed by electroporation and transformants were selected on YEP plates containing 25 µg/ml gentamycin and 50 µg/ml spectinomycin. Transient expression in tobacco epidermal cells was performed as described by Sparkes et al. [68]. YFP fluorescence was monitored 24–48 h after infiltration.

#### Confocal microscopy

A Leica TCS SP2/MP confocal laser-scanning microscope with a 20×0.7 numerical aperture water-immersion objective was used. YFP was excited at 488 nm and fluorescent emissions were measured at 518–540 nm. Chlorophyll emission was detected at 618 nm. For Figure 1B, b the settings were as follows: 10×0.7 numerical aperture water-immersion objective, YFP was excited at 512 nm, and fluorescent emissions were measured at 525 to 540 nm.

### Western Blotting

SDS-PAGE and subsequent immunoblotting were performed according to standard procedures [69]. RIN4 immunoblots were performed with affinity purified rabbit polyclonal anti-RIN4 at a concentration of 1∶1,000. AHA immunoblots were performed with rabbit polyclonal anti-AHA antisera at a concentration of 1∶5,000. The AHA antibody was raised against a C-terminal peptide of AHA2 (amino acids 852–949) [41]. Secondary goat anti-rabbit IgG-HRP conjugate (Biorad) was used at a concentration of 1∶3,000 for detection via enhanced chemiluminescence (Pierce).

### Protein Complex Purification

Protein complexes from npro*RPS2:HA* in *rps2-101c* and the *rps2-101c/rin4* negative control were purified three separate times for identification by mass spectrometry. For protein complex purifications, all steps were carried out on ice or at 4°C. 5 g of leaf tissue were ground in liquid N_2_ and resuspended in 15 ml IP buffer (50 mM HEPES, 50 mM NaCl, 10 mM EDTA, 0.2% Triton X-100, pH 7.5). Debris was removed from the lysate by centrifugation at 10,000*g*, 10 min. The supernatant was filtered through a 0.45-µm low-protein binding filter (Millipore) and incubated with 0.5 ml of affinity-purified RIN4 antisera coupled to Protein A beads (GE Healthcare). RIN4 antiserum was affinity purified according to standard protocols and 2 mg of antibody was coupled per ml of Protein A with dimethylpimelimidate [69]. The mixture was incubated end-over-end in batch format for 3 h then poured into a 20-ml glass column. Immunocomplexes were washed twice with 20 ml of wash buffer A (50 mM HEPES, 50 mM NaCl, 10 mM EDTA, 0.1% Triton X-100, pH 7.5), then twice with wash buffer B (50 mM HEPES, 150 mM NaCl, 10 mM EDTA, 0.1% Triton X-100, pH 7.5). Immunocomplexes were then washed with 5 ml of phosphate buffer (10 mM Na2PO4, 50 mM NaCl [pH6.8]) and eluted in 3×1 ml of low pH buffer (50 mM Glycine-Cl [pH2.5], 50 mM NaCl, 0.1% Triton X-100). The eluted proteins were neutralized, concentrated to a final volume of 30 µl with StrataClean resin (Stratagene), and loaded onto a single lane on a 10% SDS-PAGE gel. Proteins were run 5 mm into the separating gel and stained with colloidal coomassie blue. The resulting gel blobs were excised from the SDS-PAGE gel using a sterile blade.

### Mass Spectrometry and Protein Identification

#### Mass spectrometry

Proteins were submitted to the Genome Center Proteomics Core at the University of California, Davis, for mass spectrometry (LC MS/MS)-based protein identification. Proteins were reduced and alkylated according to previously described procedures [70], and digested with sequencing grade tryspin per manufacturer's recommendations (Promega). Protein identification was performed using an Eksigent Nano LC 2-D system (Eksigent) coupled to an LTQ ion trap mass spectrometer (Thermo-Fisher) through a New Objectives Picoview Nano-spray source. Peptides were loaded onto a Agilent nano trap (Zorbax 300SB-C18, Agilent Technologies) at a loading flow rate of 5 µl/min. Peptides were then eluted from the trap and separated by a nano-scale 75 µm×15 cm New Objectives picofrit column packed in house with Michrom Magic C18 AQ packing material. Peptides were eluted using a 60-min gradient of 2%–80% buffer B (buffer A = 0.1% formic acid, buffer B = 95% acetonitrile, 0.1% formic acid). The top ten ions in each survey scan were subjected to automatic low energy CID.

#### Database searching

Tandem mass spectra were extracted by BioWorks version 3.3. Charge state deconvolution and deisotoping were not performed. All MS/MS samples were analyzed using Mascot (Matrix Science, version 2.1.03) and X! Tandem (www.thegpm.org; version 2006.04.01.2). X! Tandem was set up to search a subset of the IPI_arabipodsis_20060916 database also assuming the digestion enzyme trypsin. Mascot was set up to search the IPI_arabipodsis_20061202 database (unknown version, 34,983 entries) assuming the digestion enzyme trypsin. Mascot and X! Tandem were searched with a fragment ion mass tolerance of 0.60 Da and a parent ion tolerance of 2.0 Da. Iodoacetamide derivative of cysteine was specified in Mascot and X! Tandem as a fixed modification. Oxidation of methionine was specified in Mascot and X! Tandem as a variable modification.

#### Criteria for protein identification

Scaffold (version Scaffold_2_01_02, Proteome Software Inc.) was used to validate MS/MS-based peptide and protein identifications. Peptide identifications were accepted if they could be established at greater than 95.0% probability as specified by the Peptide Prophet algorithm [71]. Protein identifications were accepted if they could be established at greater than 95.0% probability and contained at least two identified peptides. Protein probabilities were assigned by the Protein Prophet algorithm [72]. Proteins that contained similar peptides and could not be differentiated based on MS/MS analysis alone were grouped to satisfy the principles of parsimony.

### RT-PCR

Total RNA was extracted by a QIAGEN RNeasy Plant Mini kit and subjected to Dnase I digestion (Invitrogen). The first strand cDNA was synthesized by using 5 µg of total RNA with a cDNA synthesis kit (Promega) in a 20-µl reaction, and the reaction without reverse transcriptase served as a non-RT control. The expression level of the following genes *RIN4* (AT3G25070), *EDS1* (AT3G48090), *PAD4* (AT3G52430), *NDR1* (AT3G20600), *EFR* (AT5G20480), and *CERK1* (AT3G21630) were normalized to the expression of Actin2 (AT3G18780). RT-PCR was run for 28 cycles. The primers for all genes are listed in Table S3.

### Guard Cell Protoplast Purification

Guard cell protoplasts were isolated enzymatically from the lower leaf epidermis according to a previously described method [73]. 100–150 rosette leaves were used. Purified guard cells were visually inspected for purity by light microscopy. Guard cells were immediately used for RNA and protein extraction. Cellulose R-10 and Macerozyme R-10 were purchased from Yakult Honsha Corporation. Nylon meshes were purchased from Spectrum Laboratories, Inc.

### GUS Reporter Gene Analysis

The *AHA2:*GUS construct contained a 2,000-bp *AHA2* promoter fragment cloned into pCAMBIA 1303. *AHA2* localization in the roots of the plant lines are previously described [60].

## Supporting Information

Figure S1 **Purification of the RIN4 Complex.** (A) Affinity-purified RIN4 antibody was coupled to protein A and used to capture associated proteins in batch format. After 3 h, crude protein extract was loaded onto a glass column, contaminating proteins were removed with a high salt wash (150 mM NaCl), and the complex was eluted by low pH. (B) Anti-RIN4 immunoblot of the complex purification detecting RIN4 in the protein input and elution, but not in the column flowthrough (FT) or in the negative control (*rps2/rin4* mutant line). (C) Representative amino acid coverage of RIN4. Peptides identified in one replication are highlighted in yellow. Green indicates methionine oxidation and pPro-cmC modifications that are frequently introduced during sample processing for mass spectrometry.
(0.47 MB TIF)

Figure S2 **Expression of RIN4, AHA1_(837–950)_, and AHA2_(837–949)_ proteins in yeast.** RIN4 expression was detected by anti-RIN4 immunoblot, while AHA1/AHA2 expression was detected by anti-HA immunoblot.
(0.10 MB TIF)

Figure S3 **Vesicles isolated from wild-type plants are enriched for plasma membrane**. Plasma membrane vesicles were isolated by two-phase partitioning from the leaves of 4-wk-old wild-type Col 0 plants. H^+^-pumping activity assays were performed as described in the Materials and Methods. (A) When added at the start of the reaction, 100 µM vanadate (a plasma membrane H^+^-ATPase inhibitor) reduced pH formation 96%, while 5 µg/ml gramicidin D (an ionophore) caused the established pH gradient to completely collapse. (B) The H^+^-pumping activity was activated by 3 µM fusicoccin (FC) in the reaction solution. (C) When added after the pH formation reached steady state, 1 mM NH_4_Cl (an uncoupler) dissipated the existing pH gradient.
(0.17 MB TIF)

Figure S4 **Phenotypes of** ***AHA2*** **overexpression lines.** (A) The 35S:*AHA2_(1–837)_* overexpression line has a dwarf phenotype and displays leaflet chlorosis. Multiple independently transformed lines exhibited this phenotype. Plants are 4 wk old and were grown under the following conditions: light intensity 85 µMol/sec/m^2^, 10-h days, 24°C. (B) RT-PCR indicates that *AHA2* is overexpressed. (C) *AHA2* is expressed in guard cells. GUS staining of transgenic plants demonstrating expression of native promoter *AHA2*:GUS in *Arabidopsis* guard cells.
(0.82 MB TIF)

Figure S5 ***AHA1*** **constitutively active mutant lines are insensitive to reactive oxygen species and nitric oxide-mediated stomata closure.** The epidermal peels of Col 0, Ler, *ost2-2D*, and *ost2-1D* were floated on the 0, 0.2, 0.5 mM H_2_O_2_ (A), and 100 µM sodium nitroprusside (SNP, an NO donor) (B) for 2 h, and the stomatal aperture was recorded.
(0.14 MB TIF)

Table S1 **LC–MS/MS data for all biological replicates.** This is a summary of all the raw data from three positive and three negative replicates. Protein identification required a minimum of two peptides. These data were exported using Scaffold Viewer. Percentage indicates protein ID probability and the number of unique peptides identified per protein are in parentheses.
(0.08 MB XLS)

Table S2 **Unique peptides identified from RIN4 associated proteins by LC-MS/MS across three biological replicates.** Amino acids flanking the sequenced peptides are shown in parentheses. We were unable to differentiate between the plasma membrane H^+^-ATPases AHA1 and AHA2 in replications 2 and 3. AHA1 specific peptides are underlined in replication 1.
(0.02 MB XLS)

Table S3 Primers for RT-PCR analysis.
(0.02 MB XLS)

## Abbreviations

AHA: H^+^-ATPase
BiFC: bimolecular fluorescence complementation
CFU: colony-forming unit
ETI: effector-triggered immunity
HA: hemagglutinin
LPS: lipopolysaccharide
PAMP: pathogen-associated molecular patterns
PM: plasma membrane
PTI: PAMP-triggered immunity
R: resistance
RT-PCR: reverse-transcriptase PCR
YFP: yellow fluorescent protein
