# Supplementary figures and images for: RIN4 Functions with Plasma Membrane H+-ATPases to Regulate Stomatal Apertures during Pathogen Attack
Source: PLoS Biol. 2009 Jun 30;7(6):e1000139. doi: 10.1371/journal.pbio.1000139 (PMC2694982; doi:10.1371/journal.pbio.1000139)

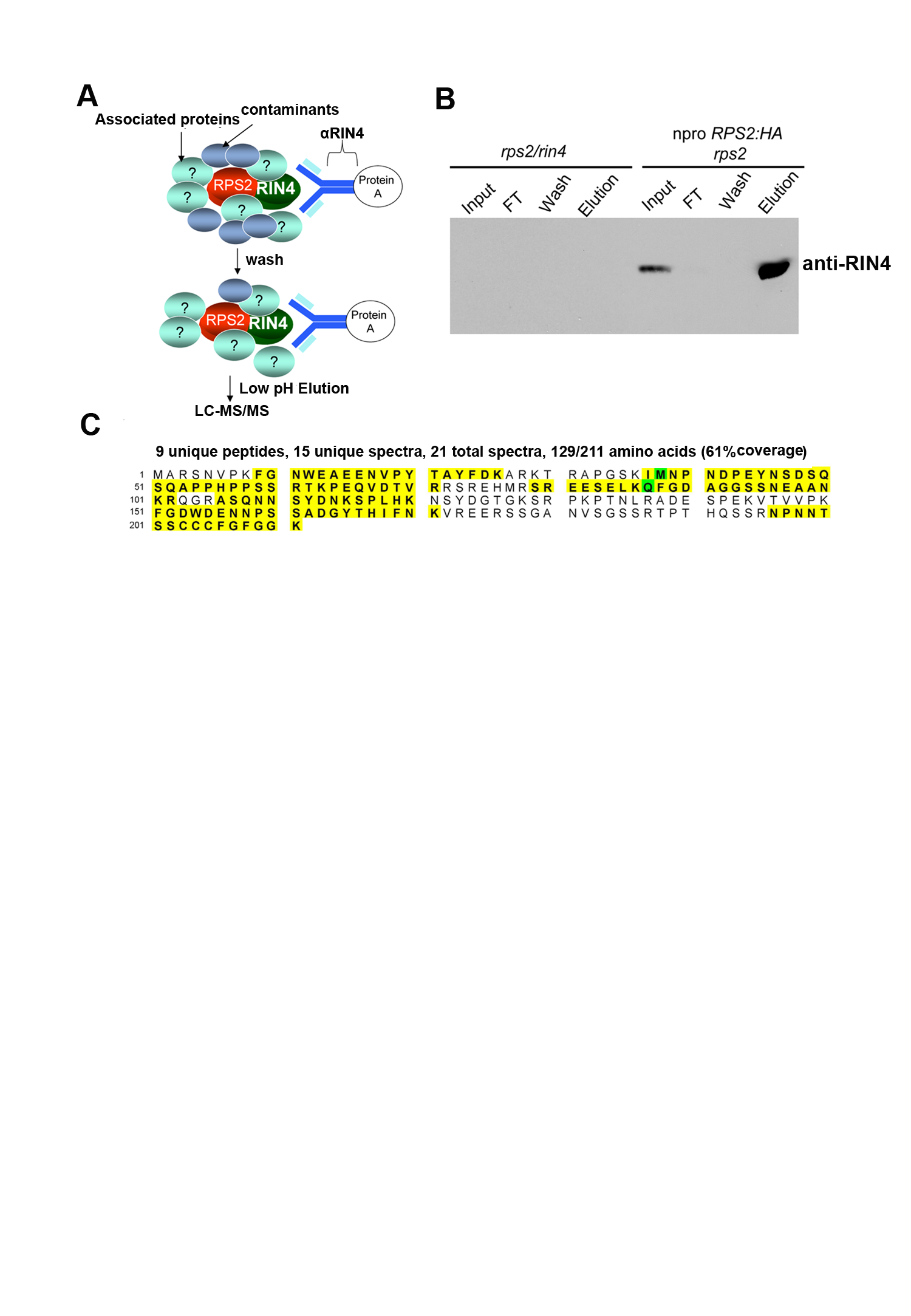

Supplement: Figure S1 — Purification of the RIN4 Complex. (A) Affinity-purified RIN4 antibody was coupled to protein A and used to capture associated proteins in batch format. After 3 h, crude protein extract was loaded onto a glass column, contaminating proteins were removed with a high salt wash (150 mM NaCl), and the complex was eluted by low pH. (B) Anti-RIN4 immunoblot of the complex purification detecting RIN4 in the protein input and elution, but not in the column flowthrough (FT) or in the negative control (rps2/rin4 mutant line). (C) Representative amino acid coverage of RIN4. Peptides identified in one replication are highlighted in yellow. Green indicates methionine oxidation and pPro-cmC modifications that are frequently introduced during sample processing for mass spectrometry. (0.47 MB TIF) [file pbio.1000139.s001.tif]

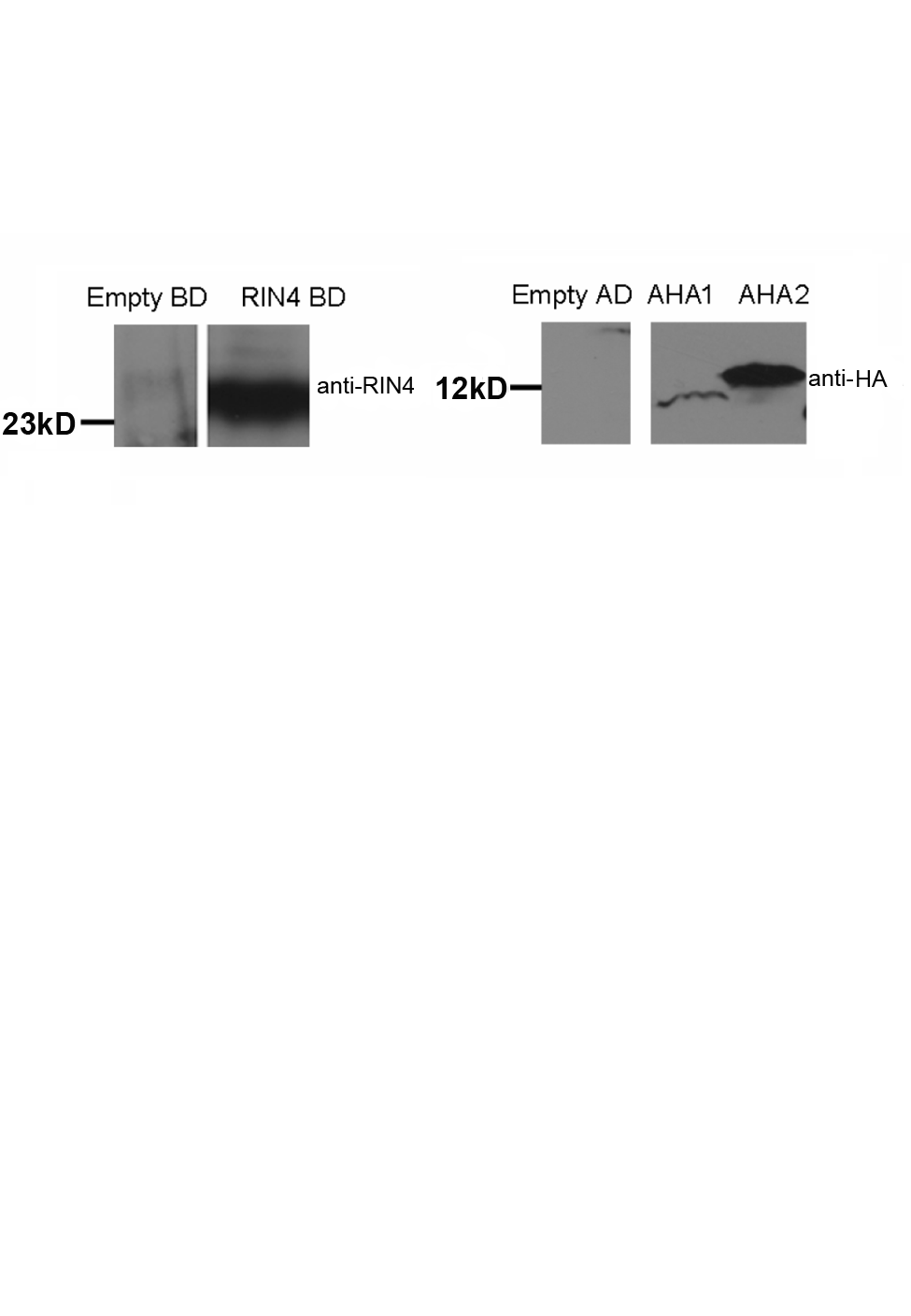

Supplement: Figure S2 — Expression of RIN4, AHA1(837–950), and AHA2(837–949) proteins in yeast. RIN4 expression was detected by anti-RIN4 immunoblot, while AHA1/AHA2 expression was detected by anti-HA immunoblot. (0.10 MB TIF) [file pbio.1000139.s002.tif]

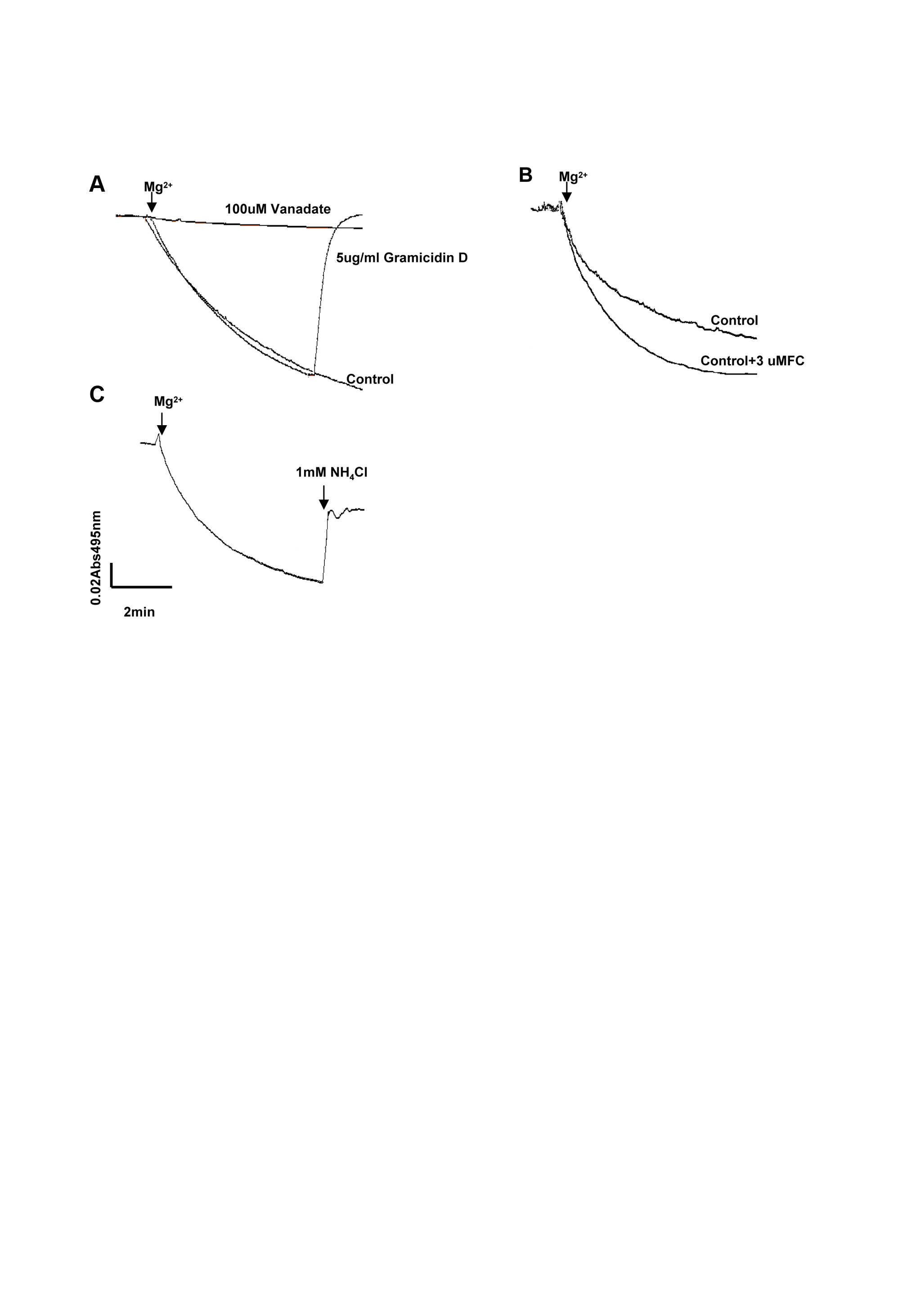

Supplement: Figure S3 — Vesicles isolated from wild-type plants are enriched for plasma membrane. Plasma membrane vesicles were isolated by two-phase partitioning from the leaves of 4-wk-old wild-type Col 0 plants. H+-pumping activity assays were performed as described in the Materials and Methods. (A) When added at the start of the reaction, 100 µM vanadate (a plasma membrane H+-ATPase inhibitor) reduced pH formation 96%, while 5 µg/ml gramicidin D (an ionophore) caused the established pH gradient to completely collapse. (B) The H+-pumping activity was activated by 3 µM fusicoccin (FC) in the reaction solution. (C) When added after the pH formation reached steady state, 1 mM NH4Cl (an uncoupler) dissipated the existing pH gradient. (0.17 MB TIF) [file pbio.1000139.s003.tif]

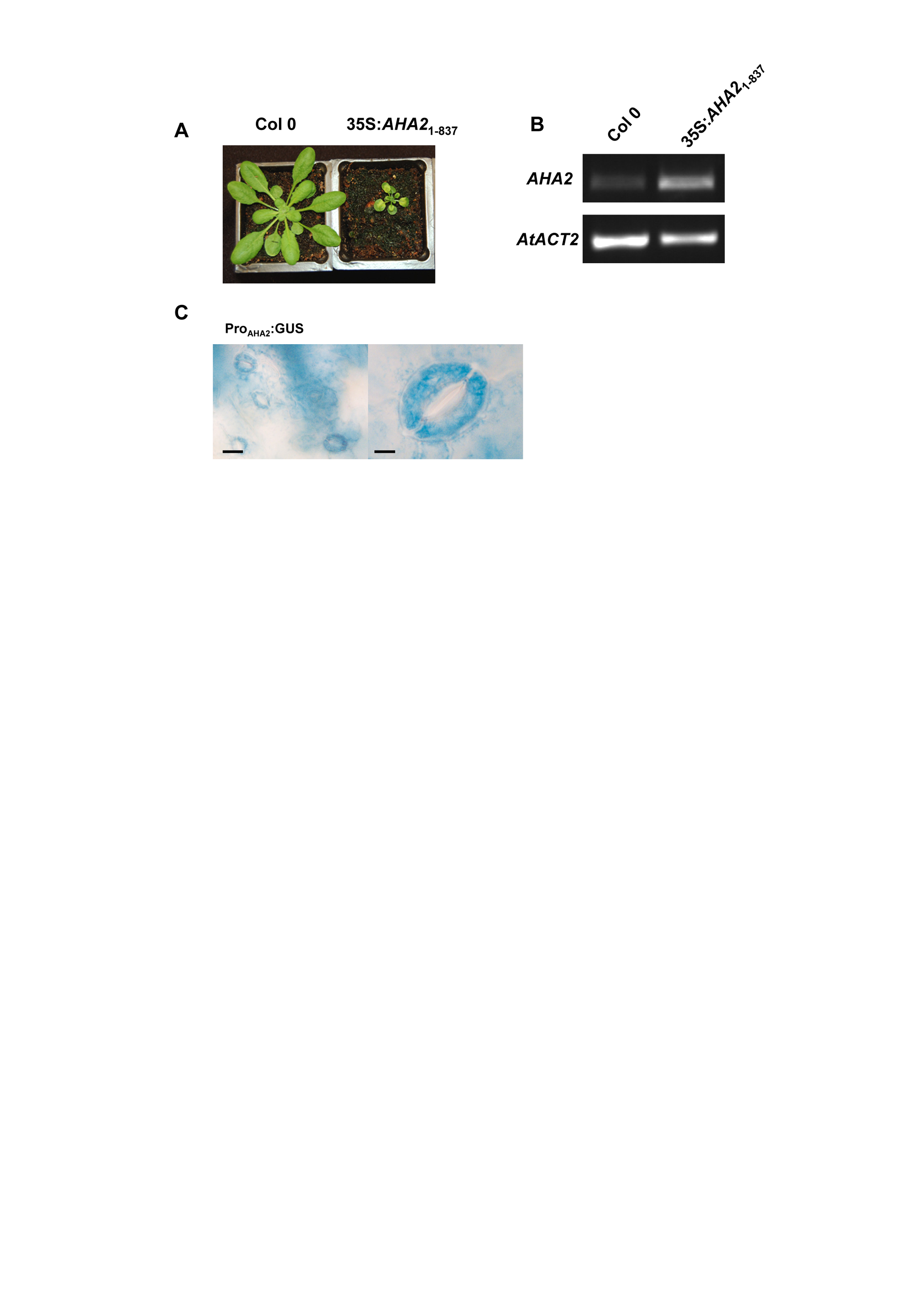

Supplement: Figure S4 — Phenotypes of AHA2 overexpression lines. (A) The 35S:AHA2(1–837) overexpression line has a dwarf phenotype and displays leaflet chlorosis. Multiple independently transformed lines exhibited this phenotype. Plants are 4 wk old and were grown under the following conditions: light intensity 85 µMol/sec/m2, 10-h days, 24°C. (B) RT-PCR indicates that AHA2 is overexpressed. (C) AHA2 is expressed in guard cells. GUS staining of transgenic plants demonstrating expression of native promoter AHA2:GUS in Arabidopsis guard cells. (0.82 MB TIF) [file pbio.1000139.s004.tif]

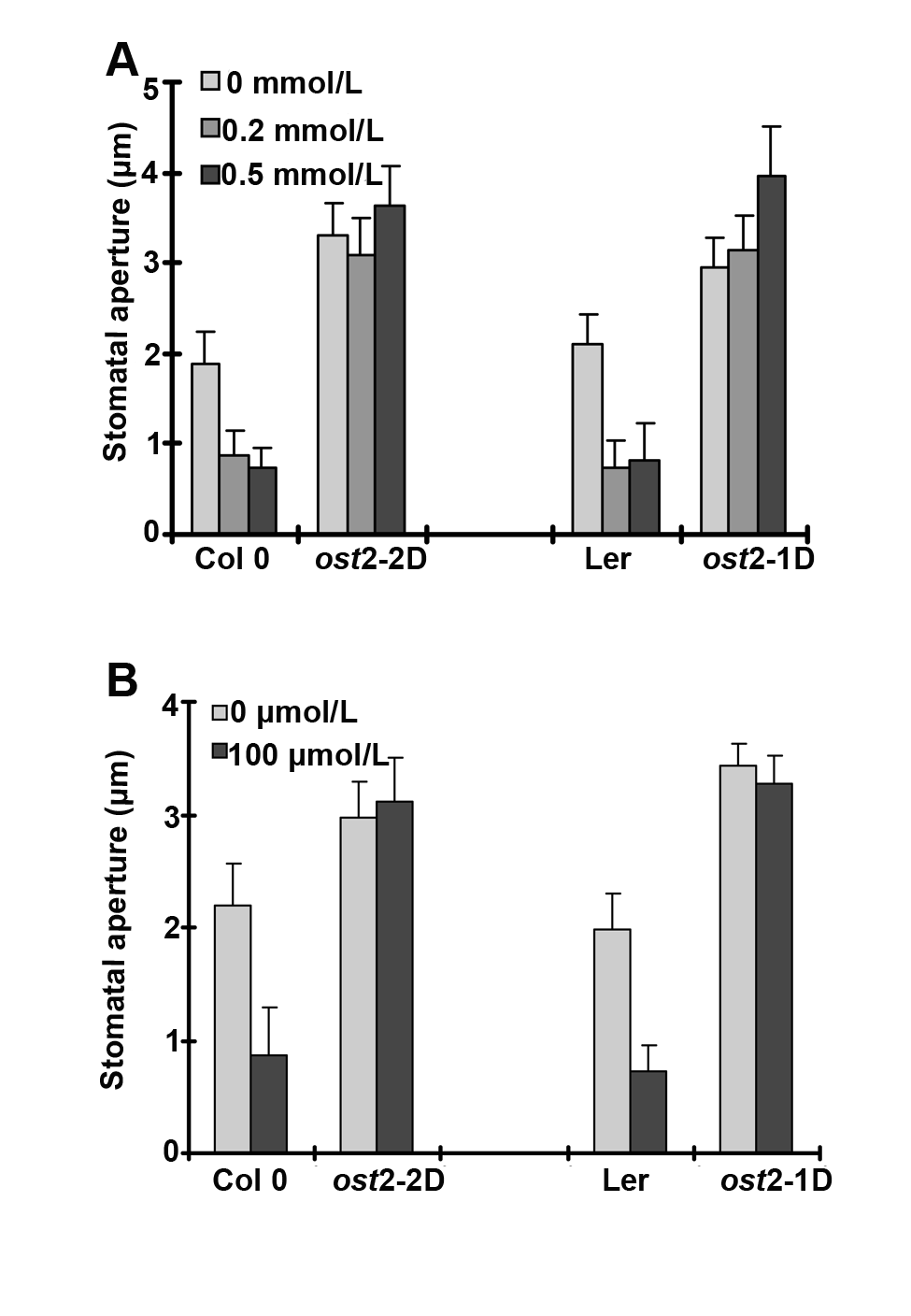

Supplement: Figure S5 — AHA1 constitutively active mutant lines are insensitive to reactive oxygen species and nitric oxide-mediated stomata closure. The epidermal peels of Col 0, Ler, ost2-2D, and ost2-1D were floated on the 0, 0.2, 0.5 mM H2O2 (A), and 100 µM sodium nitroprusside (SNP, an NO donor) (B) for 2 h, and the stomatal aperture was recorded. (0.14 MB TIF) [file pbio.1000139.s005.tif]
